# Experimental Validation of *Bacillus anthracis* A16R Proteogenomics

**DOI:** 10.1038/srep14608

**Published:** 2015-10-01

**Authors:** Zhiqi Gao, Zhiqiang Wang, Kun Zhang, Yanchang Li, Tao Zhang, Dongshu Wang, Xiankai Liu, Erling Feng, Lei Chang, Junjie Xu, Simin He, Ping Xu, Li Zhu, Hengliang Wang

**Affiliations:** 1 State Key Laboratory of Pathogen and Biosecurity, Beijing Institute of Biotechnology, 20 Dongdajie Street, Fengtai District, Beijing 100071, China; 2 State Key Laboratory of Proteomics, Beijing Proteome Research Center, National Engineering Research Center for Protein Drugs, National Center for Protein Sciences, Beijing Institute of Radiation Medicine, 27 Taiping Road, Haidian District, Beijing 100850, China; 3 Key Laboratory of Combinatorial Biosynthesis and Drug Discovery (Wuhan University), Ministry of Education, and Wuhan University School of Pharmaceutical Sciences, 185 Donghu Road, Wuchang District, Wuhan 430071, China; 4 Institute of Computing Technology, Chinese Academy of Sciences, 6 Kexueyuan Nanlu, Haidian District, Beijing 100190, China

## Abstract

Anthrax, caused by the pathogenic bacterium *Bacillus anthracis*, is a zoonosis that causes serious disease and is of significant concern as a biological warfare agent. Validating annotated genes and reannotating misannotated genes are important to understand its biology and mechanisms of pathogenicity. Proteomics studies are, to date, the best method for verifying and improving current annotations. To this end, the proteome of *B. anthracis* A16R was analyzed via one-dimensional gel electrophoresis followed by liquid chromatography coupled with tandem mass spectrometry (LC-MS/MS). In total, we identified 3,712 proteins, including many regulatory and key functional proteins at relatively low abundance, representing the most complete proteome of *B. anthracis* to date. Interestingly, eight sequencing errors were detected by proteogenomic analysis and corrected by resequencing. More importantly, three unannotated peptide fragments were identified in this study and validated by synthetic peptide mass spectrum mapping and green fluorescent protein fusion experiments. These data not only give a more comprehensive understanding of *B. anthracis* A16R but also demonstrate the power of proteomics to improve genome annotations and determine true translational elements.

*Bacillus anthracis*, the causative agent of anthrax, is a Gram-positive bacterium with two distinct morphologies, the vegetative cell and the endospore. Spores form in harsh environments and are highly resistant to environmental stresses. When the environment is suitable for growth, spores germinate and grow into new vegetative cells^1^. Therefore, spores play an important role in anthrax transmission and lead to three separate clinical presentations based on the route of entry: cutaneous, gastrointestinal, and inhalational^2^. *Bacillus anthracis* spores have been considered a potential bioweapon for years and have recently been used in bioterrorism, highlighting the need for further study of this organism.

Proteomic approaches had been extensively applied to the study of *B. anthracis* in past years^3^. Previous proteomic analysis of A16R (pXO1^+^, pXO2^−^) (GI: 749295629) whole cell extracts at exponential phase via two-dimensional (2D) electrophoresis identified approximately 300 separate proteins^4^. While the protein spots on the 2D gel allow for visualized determination of the molecular weight, isoelectric point and abundance of identified proteins, it has some shortcomings. The protein discrimination capacity of 2D electrophoresis is limited, and proteins with extreme pH values are difficult to detect. Another study in the same year identified 1,047 unique proteins from the Sterne *B. anthracis* strain^5^. Although the number of identified proteins is higher than for 2D electrophoresis, the coverage for the predicted proteome is still only about 19%. In 2007, a comparison proteomic research was carried out via 2D electrophoresis combined with Matrix-Assisted Laser Desorption/Ionization Time of Flight Mass Spectrometry (MALDI-TOF-MS) by Sung-Ha Park and colleagues and totally 1728 proteins were identified in H9401 wild type strain and 1684 in the pXO1 cured strain^6^. The relatively low coverage was suspected to be due to limitations in sensitivity, which is a major problem hindering *B. anthracis* pathogenesis and virulence research using proteomic methods.

To counter this low sensitivity, additional techniques must be applied. The rise of proteogenomics is primarily the result of the development of mass spectrometry techniques. These studies typically focus on gene annotation improvement, making use of proteomic information derived from mass spectrometry^7^. In recent years, most proteogenomic studies have been carried out via bioinformatics searches with little to no experimental validation. Combining these studies with experiment validation will further improve gene re-annotation. In this study, we combined one-dimensional (1D) gel electrophoresis and reverse-phase liquid chromatography coupled with tandem mass spectrometry LC-MS/MS (nano-UPLC-LTQ-Orbitrap Velos) to substantially increase sensitivity from our previous work. Using these techniques, higher coverage of the predicted *B. anthracis* proteome was obtained, allowing for a deeper understanding of the protein composition of this bacterium. Furthermore, we present experimental methods for validation of novel peptide fragments, which should prove a useful adjunct to future proteogenomic analyses.

## Results and Discussion

### Electrophoresis and Mass Spectrometric Identification

**Figure 1:**
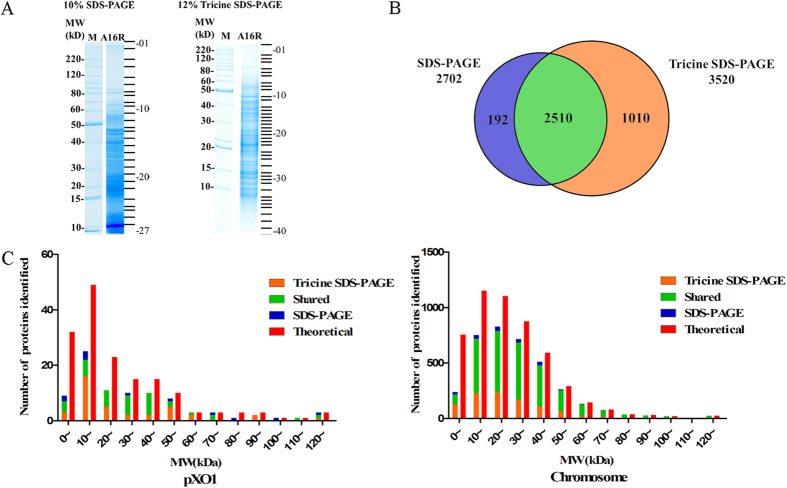
Analysis of the *B. anthracis* A16R proteome. (**A**) Protein separation by 10% SDS-PAGE and 12% Tricine SDS-PAGE. (**B**) Venn diagram of proteins identified by SDS-PAGE and Tricine SDS-PAGE. (**C**) Molecular weight distribution of identified proteins. Red bar – theoretical proteins, orange bar – Tricine SDS-PAGE, blue bar – SDS-PAGE, green bar – proteins identified by both electrophoresis approaches.

SDS-PAGE and Tricine SDS-PAGE results are shown in Fig. 1A. Samples separated better via Tricine SDS-PAGE and were more concentrated in the low-molecular-weight (MW) range using traditional SDS-PAGE. The disparity of protein band distributions between traditional and Tricine SDS-PAGE suggests that these two gel systems are complementary^8^,^9^.

A total of 3,712 proteins (3,625 proteins encoded by the chromosome and 87 encoded by plasmid pXO1) were identified via comparisons with the *B. anthracis* A16R Genbank database entry (accession NZ_CP001974), covering approximately 70.4% of the predicted proteome. Among these, 2,702 were identified from samples separated by SDS-PAGE and 3,520 from Tricine SDS-PAGE samples (Fig. 1B), indicating that Tricine SDS-PAGE was superior for protein identification. There were 192 and 1,010 proteins uniquely identified by traditional and Tricine SDS-PAGE, respectively. Most of the proteins uniquely identified by Tricine SDS-PAGE have relatively low MWs.

The identification performed by Francis *et al.* revealed 1,047 proteins^5^ using three protein separation approaches: SDS-PAGE, isoelectric focusing, and off-line two-dimensional peptide chromatography, ultimately identifying 803, 405, and 317 proteins, respectively. In this study, the combination of 1D electrophoresis and reverse-phase LC-MS/MS allows for a significantly higher degree of discrimination, suggesting this technique is more robust.

### Physical and Chemical Property Distributions for Identified Proteins

**Figure 2:**
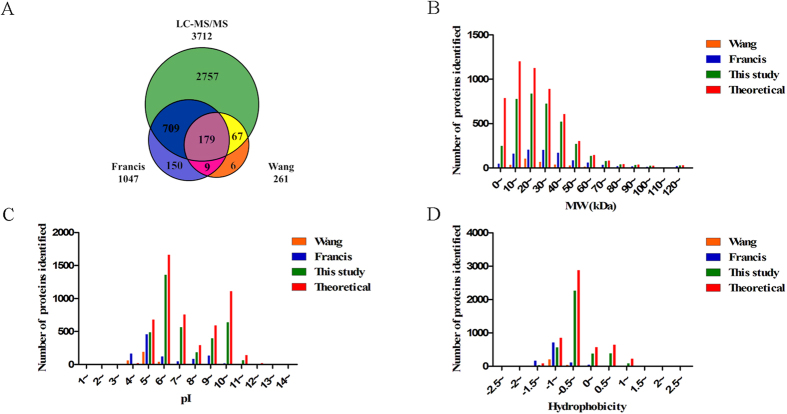
Comparison of proteome data sets. (**A**) Venn diagram of unique and shared non-redundant identified proteins between the three datasets. (**B**) Molecular weight distribution. (**C**) p*I* distribution. (**D**) Hydrophobicity distribution. Red bar –theoretical proteins; green bar – this study; blue bar – Francis *et al.*, orange bar – Wang *et al.*

The MW distribution of identified proteins was different between the two separation approaches (Fig. 1C). Chromosomal proteins were more likely to be identified after Tricine SDS-PAGE separation and approximately 37% more proteins with MWs between 10 and 20 kDa were identified by Tricine SDS-PAGE than by traditional SDS-PAGE. A total of 244 and 118 proteins with MWs lower than 10 kDa were identified by Tricine and traditional SDS-PAGE, respectively, further emphasizing the advantage of Tricine SDS-PAGE for protein separation. These results are a substantial improvement in protein identification compared with the 261 proteins identified by Wang via 2D electrophoresis and the 1,047 proteins identified by Francis (Fig. 2A).

The MW, pI, and hydrophobicity of identified proteins were also compared between this study and two previous proteomics studies in *B. anthracis* (Fig. 2B–D). The p*I* range of identified proteins was 3.40–12.61 (Fig. 2C). About 80% proteins with p*I*s between 5 and 6 in A16R Genbank database were identified, which was the highest among the various p*I* intervals.

### Functional Analysis for Identified Proteins

#### Regulatory Proteins

Five important regulatory proteins in the *B. anthracis* regulatory network^10^ were identified, including the global regulatory protein AtxA, the S-layer expression regulatory protein PagR, and the sporulation regulatory protein Spo0A (Supplementary Table S1). The most important regulatory protein, AtxA (encoded by the *atxA* gene), is a global regulator in *B. anthracis* and regulates the expression of hundreds of genes, both chromosomal and plasmid^10^. Our results reveal 16 unique peptides matching AtxA via Tricine SDS-PAGE but only nine via traditional SDS-PAGE, despite AtxA having a MW of approximately 56 kDa. As AtxA was not identified by either Wang or Francis, this also highlights the need for increased sensitivity for regulatory protein detection. PagR, another identified regulatory protein whose MW is approximately 12 kDa, can suppress transcription of *sap* by binding the promoter area of the *sap* gene. Other detected proteins include CodY, which may be associated with AtxA degradation, Spo0A, which initiates sporulation, and σ^H^, whose function is currently unknown^11^,^12^.

The expression of PlcR, an important regulatory protein in *B. cereus*^13^,^14^ but encoded by a pseudogene in *B. anthracis*, was also detected. In *B. anthracis*, there is a mutation in the *plcR* gene that might lead to a loss of function. Our proteogenomic analysis reveals that the PlcR is expressed, although a lack of identified peptides belonging to the C-terminal end of PlcR suggests early termination compared with *B. cereus*.

### *Sporulation- and Germination-associated Proteins*

The identified spore-associated proteins, including sporulation-associated proteins, spore germination-associated proteins, and spore component proteins, are listed in Supplementary Table S1. A16R_23730 and A16R_44450 were revealed as high-abundance proteins based on the mass spectra counts. Because the samples were harvested during the early exponential phase, it is reasonable that proteins associated with sporulation control were continuously expressed. Another sporulation protein, A16R_39640, annotated as a stage V sporulation protein with a MW of about 8.86 kDa, was also revealed as a high-abundance protein. The expression of this protein in the exponential phase indicates a potential important function other than sporulation, which is worthy of further analysis in the future.

It has been reported that some sporulation-associated proteins are expressed in the plateau phase prior to sporulation^15^. Our data identified several proteins from different sporulation stages, although most of these had few spectra counts, indicating low abundance. The identification of these proteins may be because of vegetative cell heterogeneity, as some cells may have sporulated earlier.

### *S-layer Proteins*

The S-layer is a putative virulence factor composed of various ultrastructural S-layer proteins that completely covers the cell surface. In total, 14 S-layer proteins were identified in this study. In *B. anthracis*, two S-layer proteins, the surface array protein (Sap) and extractable antigen 1 (EA1), are the most abundant. Our findings confirm that in the early exponential phase, these two S-layer proteins are some of the most abundantly expressed proteins. S-layer proteins in *B. anthracis* have a dynamic interchange: Sap is first expressed on the cell surface, followed by EA1, which displaces Sap and allows its release to the culture supernatant^16^. As both were detected, sampling may have occurred during this process of dynamic interchange in the early exponential phase. Besides the Sap and EA1 proteins, A16R_33930 (GBAA_3338 in *B. anthracis* Ames Ancestor^17^) showed a relatively high spectra count indicating a relatively high abundance.

### *Hypothetical Proteins*

A total of 338 proteins annotated as hypothetical were proved to be expressed. Among these, 323 proteins are encoded by chromosomal genes and 15 by plasmids (Supplementary Table S4).

### *Unidentified Proteins*

Proteins may not have been identified for several reasons. First, proteins may not have been expressed under the culture conditions we analyzed, particularly proteins specific for spores. Second, despite using two protein separation approaches, proteins with small molecular weights may be lost during electrophoresis, particularly those with MWs less than 3 kDa. Third, proteins with very low abundance may fall below the detection limit of our assay. Finally, proteins with special properties, such as high hydrophobicity and a lack of enzymatic cleavage sites, may not be detected. For example, the 18.5-kDa collagen-like glycoprotein BclA in *B. anthracis*^18^, which contains only two lysines and no serines, produces peptides too large to be detected by mass spectrometry after trypsin digestion.

### Sequencing Error Correction and Novel Gene Detection

#### Sequencing Error Correction

To determine possible sequencing errors, the list of 5,584 proteins identified by searching the six-reading-frame (6F) database was compared to the A16R Genbank database, detecting a total of 2,306 unpaired proteins. When the unpaired unique proteins from the 6F database were compared to protein databases of other *B. anthracis* strains, a total of 149 proteins were matched to 72 proteins of *B. anthracis* Ames Ancestor (Supplementary Table S2). Given the highly conservation of *B. anthracis*, these results were suspected to be due to genomic sequencing errors in the annotated *B. anthracis* A16R genome.

**Figure 3:**
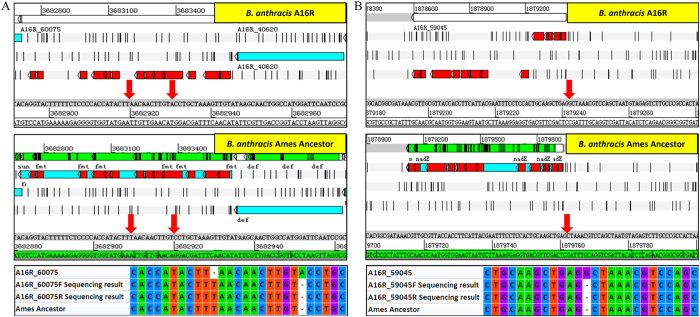
Validation of genome sequencing errors. Upper panel: Genomic annotation in *B. anthracis* A16R; Middle panel: Genomic annotation in *B. anthracis* Ames Ancestor; Lower panel: Sequencing validation. The red parts indicate the location of peptides identified in this study. (**A**) Sequencing errors causing annotation of open reading frames as pseudogenes with premature termination. (**B**) Sequencing errors causing frameshifts.

A total of 8 out of the 149 regions corresponding to the proteins were randomly selected for validation by polymerase chain reaction (PCR) and re-sequencing, with the primers used listed in Supplementary Table S3. Both single-base deletion and insertion errors were found (Fig. 3 and Supplementary Fig. S3). These sequencing errors mistakenly caused the annotation of the open reading frames as pseudogenes with premature termination (as shown in Fig. 3A) or frameshifts (as shown in Fig. 3B) in the *B. anthracis* A16R Genbank database. Our results provide experimental proof for expression of the integrated gene.

### *Novel Peptide Fragments*

**Figure 4:**
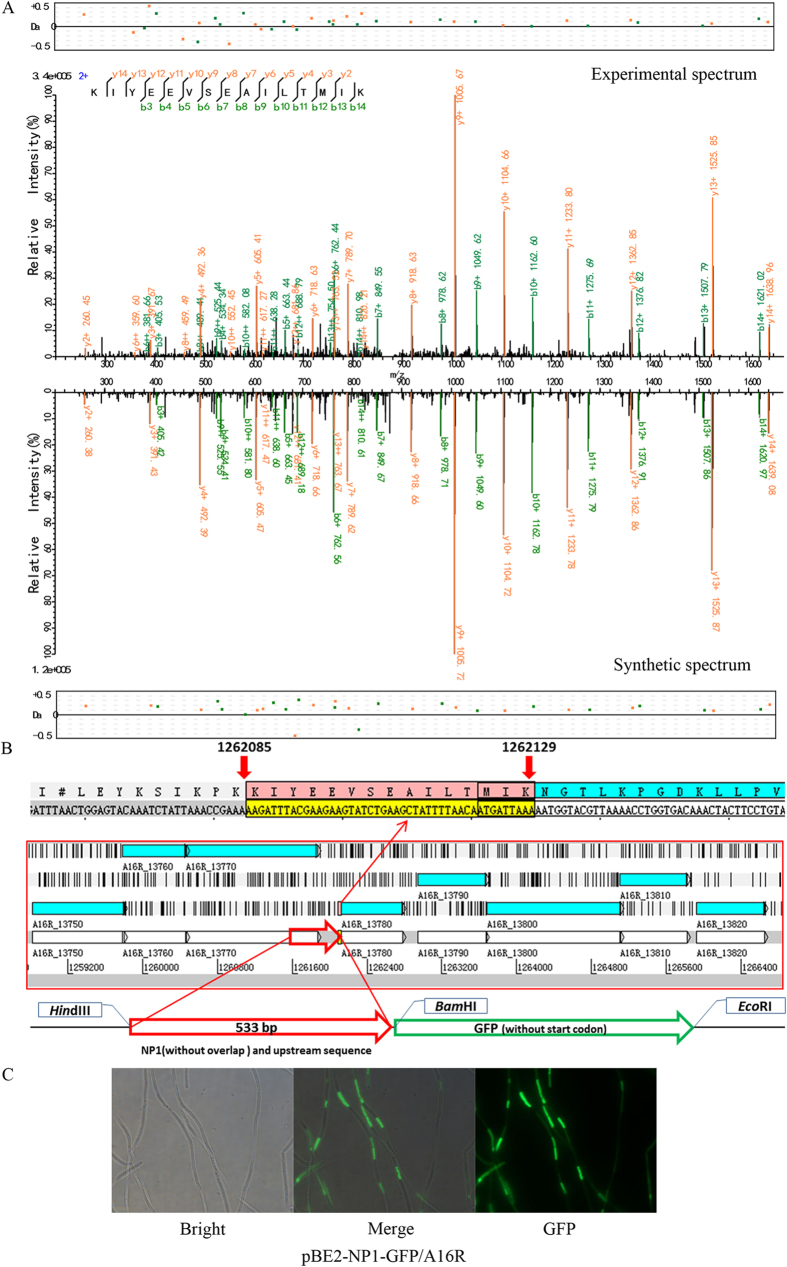
Validation of peptide fragment NP1. (**A**) Synthetic peptide mass spectrum mapping of NP1. (Top) Spectra filtered by proteogenomic analysis. (Bottom) Spectra of synthesis peptide. (**B**) The encoded region of NP1. (**C**) Validation of NP1 by GFP fusion experiment.

**Figure 5:**
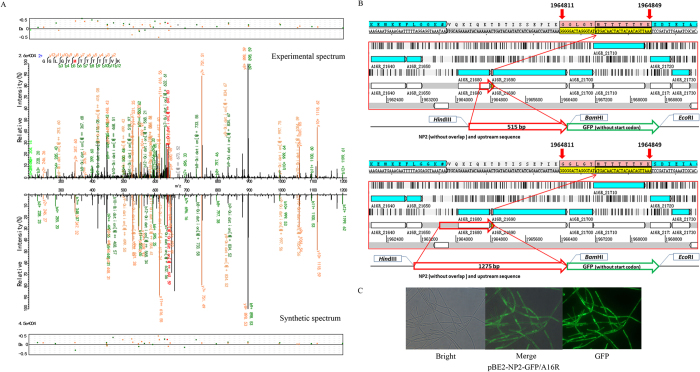
Validation of expression of peptide fragment NP2. (**A**) Synthetic peptide mass spectrum mapping of NP2. (Top) Spectra filtered by proteogenomic analysis. (Bottom) Spectra of synthesis peptide. (**B**) The encoded region of NP2. The 515-bp (top) and 1275-bp (bottom) fragments were respectively cloned and fused with the *gfp* gene. (**C**) Validation of NP2 by GFP fusion experiment. No green fluorescence was observed when the 515-bp fragment was inserted to the vector.

**Figure 6:**
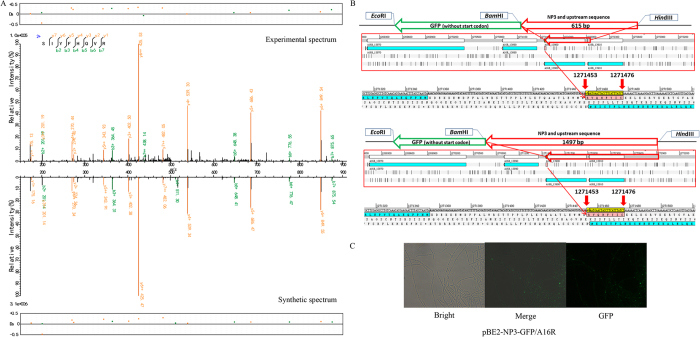
Validation of peptide fragment NP3. (**A**) Synthetic peptide mass spectrum mapping of NP3. (Top) Spectra filtered by proteogenomic analysis. (Bottom) Spectra of synthesis peptide. (**B**) The encoded region of NP3. The 615-bp length (top) and 1497-bp length (bottom) sequence were respectively cloned and fused with the *gfp* gene. (**C**) GFP signal validation of NP3. No fluorescence was observed when the 515-bp fragment was inserted into the vector.

Four peptide fragments that were not present in the annotation of any *B. anthracis* strain were discovered by our proteogenomic analysis (Supplementary Fig. S1). Further analysis of adjacent genetic elements revealed that three of these peptide fragments (NP1–3) may indicate new start codons for previously annotated proteins and one peptide fragment (NP4) may indicate a new gene (Table 1). NP1 is an upstream extension of the annotated protein “GntR family transcriptional regulator” (Fig. 4). NP2 is an upstream extension of the “formate-tetrahydrofolate ligase” protein (Fig. 5). The annotated start codon of the gene downstream to NP3 was different between *B. anthracis* H9401 (H9401_1240, Dehydratase) (Supplementary Fig. S7C) and the other five examined strains (in Ames Ancestor, it is annotated as GBAA_1326, maoC family protein) (Supplementary Fig. S7B). These peptides were synthesized and tested via LC-MS/MS. The spectra of NP1–3 revealed a high agreement with the proteomic results both in the peptide fragments distribution and the relative intensity between the peaks (Fig. 4, 5, 6). However, the spectrum of the synthesized NP4 is different from that obtained from the bacteria, suggesting this is a false-positive result (Supplementary Fig. S4).

Green fluorescent protein (GFP) fusion experiments were also performed to experimentally validate the translation of these four peptide fragments. Green fluorescence was observed when recombinant plasmids for promoter activity validation of the former three peptides were introduced into *B. anthracis* A16R (Fig. 4C, 5C and 6C), but no fluorescence was observed with recombinant plasmids for validation of NP4 or the negative control vector pBE2-GFP (Supplementary Fig. S4-5). A lack of GFP expression and poor spectrogram matches indicates that NP4 was a spurious result. Particularly, compared to NP1 and NP2, the fluorescence intensity of NP3 was weaker and focal, which may be because of low protein expression and protein polarity.

To determine whether these novel peptides represented a new start codon for previously predicted genes or a frameshift of the upstream-annotated gene, western blot analysis (Supplementary Fig. S8) was performed to analyze the MWs of the fusion proteins expressed by the recombinant plasmids. The MWs of NP1-GFP and NP2-GFP fusion proteins are slightly larger than that of GFP, indicating that these peptides (NP1 and NP2) are translated independently from the upstream genes although they are transcribed together. Multiple bands appeared when the NP3-GFP fusion protein was blotted with an anti-GFP antibody, suggesting that this protein likely has a much larger MW than GFP, suggesting that the fusion protein may be the product of read through of the upstream gene. The difference in green fluorescence strength between NP3-GFP and the other two fusion proteins indicates that this peptide belongs to a gene product with a frameshift caused by ribosome slipping and proteolysis.

The expression of GFP indicates that the three peptide fragments were genuinely translated, suggesting that the corresponding downstream genes should be reannotated to alternative start codons. No “ATG”, “GTG”, or “TTG” was found between NP1 and its nearest stop codon upstream (Fig. 4C). Green fluorescence was observed when the 533-bp fragment upstream, including the region encoding the first 12 amino acids of NP1, was cloned and fused with the *gfp* gene. There is a leucine encoded by “CTG” upstream from the peptide fragment with a “GAGG” ribosome binding site (RBS) 9 bp upstream. Previous studies have reported that the codons CTG (leucine), ATT (isoleucine), ATA (isoleucine), and ATC (isoleucine) can also function as translation initiators in *Escherichia coli*^19^,^20^. Whether these function similarly in *B. anthracis* is currently unknown.

For NP2, the upstream valine coded by “GTG” with an “AGGAGG” RBS 7 bp upstream may represent the true start codon. In some *B.* cereus strains, the annotation of translation initiators of “formate-tetrahydrofolate ligase” is similar to our proposal (*B. cereus* B4264, GI: 218230750). The genome sequences upstream of the NP2 are different between *B. anthracis* and *B. cereus* (Supplementary Fig. S6), which may reflect genome sequencing errors or genetic diversity, either of which could lead to the annotation differences (Fig. 5C). Moreover, no green fluorescence was observed when a 515-bp fragment upstream, including the encoded region of the first five amino acids of NP2 was inserted into the vector; however, when a 1275-bp fragment was inserted into the vector, including the upstream annotated gene “A16R_21680” and its promoter site, green fluorescence was observed, suggesting that the expression of *gfp* may be because of a multi-cistron structure.

The NP3 peptide expression suggests that the reading frames between A16R_13890 and A16R_13900 (PhaP protein) may overlap. However, no overlap was found in the current genome annotations of all *B. anthracis* strains, although the start codon annotation is different between *B. anthracis* H9401 and other *B. anthracis* strains (Supplementary Fig. S7). Similar to NP2, green fluorescence was observed only when the longer fragment of the upstream gene with its promoter site was inserted to the vector. No apparent start codon or RBS was found upstream of the NP3.

Based on these results, these methods are useful for novel gene discovery and genome annotation correction. Start codon annotation errors might be due to unusual start codons or other translation mechanisms and require further analysis. These techniques may also be useful in creating an annotated data set as a reference for adjustment of automated gene annotation software. Despite its utility, the combination of 1D electrophoresis and reverse-phase LC-MS/MS has some limitations that may cause low-molecular-weight proteins to be lost. Low-molecular-weight protein collection methods, such as ultrafiltration, may be useful to increase sensitivity. Considering the spore-forming nature of *B. anthracis*, the proteomic component is different in different growth phases. Therefore, the higher sensitivity of these techniques would be more useful if coupled to comparison of different bacterial growth phases.

The A16R strain has been used for vaccine development in China. The semi-quantitated dataset of protein abundance in this study would be valuable for the development of next generation live attenuated vaccines. It is the first attempt for application of proteogenomics on *B. anthracis* to find new genes (or isoforms) and highlight sequencing errors. A relatively large reference dataset for this organism has been constructed and future studies may expand this further by collecting similar proteogenomic data under different growth conditions. Thus, this study might be a starting point for more advanced comparative proteogenomic studies in *B. anthracis*.

## Materials and Methods

### Sample Preparation and Protein Separation

*Bacillus anthracis* A16R strain is a vaccine strain that was obtained by mutagenesis of wild-type *B. anthracis* A16. A single colony was picked from Luria Bertani (LB) solid medium and inoculated into 5 mL of LB broth. Cells were grown aerobically at 37 °C with shaking for 12 h. The culture was inoculated into 50 mL LB broth at an initial optical density (OD) at 600 nm of 0.1, then grown under the same conditions for approximately 4 h until the final OD_600_ reached 3.0. This culture was inoculated into 100 mL of LB broth with an initial OD_600_ of 0.1 and grown under the same conditions for approximately 4 h. Cells in early exponential phase (OD_600_ reached 3.0) were harvested by centrifugation at 4 °C and 7,000 × g for 10 min. The pellet was washed twice with a low-salt washing buffer (3 mM KCl, 68 mM NaCl, 8 mM NaH_2_PO_4_, and 1.5 mM KH_2_PO_4_, kept at 4 °C) and collected via centrifugation at 4 °C and 7,000 × *g* for 5 min. The pellet was resuspended in 5 mL of lysis solution (7 M urea, 2 M thiourea, 1% DTT, and a half of a protease inhibitor cocktail tablet, Roche, Switzerland) and disrupted by ultra-sonication at 25% amplitude for 15 min at 0 °C. The resulting suspension was centrifuged at 18 °C and 40,000 × *g* for 30 min and the supernatant collected. Protein concentration was determined using the 2-D Quant Kit (GE Healthcare, Piscataway, NJ, USA) and the supernatant was stored at −70 °C for further analysis.

Extracted protein samples were boiled for 5 min and alkylated using 20 mM iodoacetamide in the dark for 30 min after cooling to room temperature. Approximately 120 μg of each protein sample was mixed with 5× SDS loading buffer and separated via either traditional (10%) or Tricine (12%) SDS-PAGE. Gels were stained with Coomassie blue G250 containing 0.1% Coomassie, 20% methanol, and 5% acetic acid for 3 h to stabilize the protein bands.

### In-gel Digestion

Protein lanes from the traditional (27 pieces) and Tricine (40 pieces) gels were excised (Fig. 1A) based on molecular weight and local protein amount. Each band was put into an Eppendorf tube, minced into 1-mm^3^ cubes, and destained with 30% acetonitrile and 35 mM ammonium bicarbonate until colorless. Gel pieces were shrunken with pure acetonitrile and dried by vacuum concentration. A total of 50 mM ammonium bicarbonate was added to each tube in enough volume to cover the gel pieces, 40 μL of 12.5 ng/μL trypsin was added to each tube, and tubes were placed on ice for 40 min. All tubes were incubated at 37 °C for 12 h and centrifuged at 12,000 × g for 1 min. Peptides were extracted from each supernatant as follows: (1) 40 μL extraction buffer (5% formic acid, 50% acetonitrile) was added to each tube. (2) Tubes were centrifuged at 12,000 × g for 1 min, and rested for 4 min at room temperature. Both (1) and (2) were repeated four times and all supernatants were collected into new tubes. (3) Each tube had 40 μL of 100% acetonitrile added with 20 min of vortexing. (4) Tubes were centrifuged for 1 min at 12,000 × g for 1 min and rested for 4 min at room temperature. Steps (3) and (4) were repeated four times and all supernatants were collected. The resulting peptides were dried via vacuum concentration.

### LC-MS/MS Analysis

The UPLC system (nanoAcquity Ultra Performance LC, Waters Corporation, Milford, MA, USA) was used for peptide separation. The UPLC column is a 75-μm inner diameter (id) ×15-cm-long fused-ailica capillary tube packed with RepoSil-Pur C_18_-AQ 1.9 μm resin (Dr. Maisch, Ammerbuch-Entringen, Germany). Samples were loaded at 0.3 μL/min and eluted for 40 min using a 5–40% ACN fraction-optimized nonlinear gradient in 0.1% formic acid. Eluted peptides were analyzed using a LTQ-Obitrap Velos mass spectrometer (Thermo Fisher Scientific, Waltham, MA, USA). Survey scans were performed at a resolution of 30,000 at target values of 1,000,000 ions in the Orbitrap analyzer with maximum allowed fill times of 150 ms over a mass range of 300–1,600 m/z. The 20 most intense precursor ions were chosen for collision-induced fragmentation. A total of 5,000 ions were accumulated over a fill time of 25 ms and fragmented by wideband activation on each scan. Exclusion of precursor ion masses over a time window of 30 s was used to suppress repeated fragmentation of peaks^21^,^22^.

### 6F Database Construction

The 6F database was constructed based on all possible proteins from all six open reading frames in the genome, with all sequences having 10 or more amino acids between two stop codons regarded as a potential protein. The final 6F database contained 299,507 entries.

### Data Process and Analysis

All spectra were searched against the *B. anthracis* A16R Genbank database using the pFind^23^,^24^,^25^ (version 2.8.0.1, Bioinformatics Group, Institute of Computing Technology, Chinese Academy of Sciences) search engine. Results were filtered by pBuild software (2.0.0.8) using a target-decoy strategy. The decoy database was created by reversing the order of the amino acid sequence for each protein in A16R Genbank database^26^. The false discovery rate (FDR) at the peptide spectrum match (PSM) level was estimated as the ratio of the number of decoy matches to that of target matches and the FDR threshold was set as 1%^27^. The peptide length was required to be between 6 and 60. Proteins with at least one unique peptide were allowed. Both complete and partial trypsin digestion peptides were allowed with up to two miscleavages. Tolerance of precursor mass was 20 ppm and fragment mass deviation was 0.5 Da. Carbamidomethylation of cysteine (+57.0215 Da) and oxidation of methionine (+15.9949 Da) were set as fixed and variable modifications, respectively.

### Proteogenomic Analysis

All spectra were searched against the 6F database using pFind tools with parameters set as described above for A16R Genbank database searches. Peptide fragments and proteins that were not identified in the A16R Genbank database were further analyzed via alignment with the National Center for Biotechnology Information database using protein-protein BLAST. Peptide fragments with no homology to other *B. anthracis* genomes were manually checked. Spectra with a continuous series of at least 3 y^+^ or b^+^ ions and the intensity of base peak not less than 10,000 were reserved when manual checking.

### Peptide Fragment Synthesis

To further confirm the accuracy of the LC-MS/MS detection, peptide fragments with no homology to annotated *B. anthracis* genes were synthesized and analyzed by LC-MS/MS with the same parameters as previously described. Results were compared with prior results; detection should be similar, and divergent results were considered spurious and discarded.

### Promoter Activity Validation

The *gfp* gene, coding for GFP, was used as a reporter gene in constructing vectors to determine DNA fragment promoter activity. Green fluorescence would be observed if the DNA fragment is expressed. (See supporting information for additional details.)

### Western blot analysis

Strains with recombinant plasmids were cultured to the exponential phase and 1-mL bacterial suspension was collected by centrifugation at 12,000 × g for 1 min at room temperature. The pellet was suspended in 40 μL ddH_2_O and 40 μL 2× SDS sample buffer (25 mM Tris pH 6.8, 0.8% SDS, 4% glycerol, 2% 2-mercaptoethanol, and 0.005% bromophenol blue). Samples were boiled for 15 min and separated by 12% SDS-PAGE. Proteins separated by SDS-PAGE were electrotransferred to a PVDF membrane under a constant 15V. The PVDF membrane was blocked in TBST (0.3% Tris, 0.88% Nacl, 0.02% KCl, 0.05% Tween20, pH = 7.4) containing 5% dried skim milk for 1 h at 37 °C. The membrane was incubated with a rabbit anti-GFP antibody (anti-GFP antibody ab6556, Abcam, UK) diluted 1:1000 in TBST for 1 h at room temperature. The membrane was then washed in TBST and incubated with a goat anti-rabbit IgG antibody diluted 1:1000 in TBST for 1 h at room temperature. Immunoreactivity was visualized using an imaging apparatus (Tanon 5200, China) with enhanced chemiluminescence solution (SuperSignal West Pico Stable Peroxide Solution, PC 199723; SuperSignal West Pico Luminol Enhance Solution, PC 199939, Thermo, USA).

## Additional Information

**How to cite this article**: Gao, Z. *et al.* Experimental Validation of *Bacillus anthracis* A16R Proteogenomics. *Sci. Rep.* **5**, 14608; doi: 10.1038/srep14608 (2015).

## Supplementary Material

Supplementary Information

## Figures and Tables

**Table 1:** Peptide fragments identified by proteogenomic analysis.

Name	Peptide fragment	6F database	Strand	Location in *B. anthraci*s A16R
NP1	K. KIYEEVSEAILTMIK. N	A16R_3_26546	+	1262085–1262129
NP2	K. GGLGYMTTTTTVK. S	A16R_3_41393	+	1964811–1964849
NP3	K. SIYFHQVR. K	A16R_rv3_79827	−	1271453–1271476
NP4	*IMSPVASLIR. T	A16R_rv2_40766	−	3255309–3255338
