# Supplementary material for: Experimental Validation of Bacillus anthracis A16R Proteogenomics
Source: Sci Rep. 2015 Oct 1;5:14608. doi: 10.1038/srep14608 (PMC4589699; doi:10.1038/srep14608)
Supplement: Supplementary Information [file srep14608-s1.pdf]

Supporting Information

Experimental Validation of *Bacillus anthracis* A16R Proteogenomics

Zhiqi Gao[1, +], Zhiqiang Wang[2, 3, +], Kun Zhang[4], Yanchang Li[2], Tao Zhang[2], Dongshu Wang[1], Xiankai Liu[1], Erling Feng[1], Lei Chang[2], Junjie Xu[1], Simin He[4], Ping Xu[2, 3,*], Li Zhu[1,*], Hengliang Wang[1,*]

[+] These authors contributed equally to this work.

[1]State Key Laboratory of Pathogen and Biosecurity, Beijing Institute of Biotechnology, 20 Dongdajie Street, Fengtai District, Beijing 100071, China

[2]State Key Laboratory of Proteomics, Beijing Proteome Research Center, National Engineering Research Center for Protein Drugs, National Center for Protein Sciences, Beijing Institute of Radiation Medicine, 27 Taiping Road, Changping District, Beijing 102206, China

[3]Key Laboratory of Combinatorial Biosynthesis and Drug Discovery (Wuhan University), Ministry of Education, and Wuhan University School of Pharmaceutical Sciences, 185 Donghu Road, Wuchang District, Wuhan 430071, China

[4]Institute of Computing Technology, Chinese Academy of Sciences, 6 Kexueyuan Nanlu, Haidian District, Beijing 100190, China

1. Promoter activity validation

The *Bacillus subtilis* and *Escherichia coli* shuttle plasmid (pBE2) was used for vector construction and *gfp* coding for green fluorescence protein (GFP) as a reporter gene. First, the *gfp* fragment was inserted into the pBE2 vector. Then, the DNA fragment upstream of the potential CDS corresponding to the identified peptide fragment was inserted upstream of the *gfp* fragment (Figures 2B, 3B, and 4B). The start codon of *gfp* gene was eliminated to ensure that the observation of the green fluorescence signal was due to transcription and translation of the inserted DNA fragment. The vector was constructed as follows (Figure S2):

(1) The restriction enzymes *Bam*HI and *Eco*RI were chosen to linearize the pBE2 vector. A mixture of 20 μL of pBE2, 13 μL ddH$_2$O, 4 μL buffer solution, and 1.5 μL each of the *Bam*HI and *Eco*RI restriction enzymes were incubated at 37°C for 30 min. Linearized plasmids were recovered using a DNA purification kit.

(2) The *gfp* fragment minus the start codon was cloned by PCR using 5 μL of PCR buffer, 4 μL dNTP, 37 μL ddH$_2$O, 1 μL of each primer, 1 μL template, and 1 μL high fidelity polymerase. The PCR conditions were 94°C for 10 min, followed by 30 cycles of 94°C for 30 s, 55°C for 30 s, and 72°C for 60 s. The reaction was incubated at 72°C for 10 min for fragment extension. PCR products were then purified and recovered using a DNA purification kit (CWBIO, China).

(3) The *gfp* DNA fragment was cut using *Bam*HI and *Eco*RI as per step 1 and recovered.

(4) The ligation reaction was performed using 1 μL of vector, 7 μL of *gfp* fragments, 1 μL of buffer, and 1 μL ligase enzyme, and incubated at 22°C for 20 min. Recombinant plasmids

were screened and named pBE2-GFP.

(5) Peptide associated fragments were inserted to the pBE2-GFP vector using *Hin*dIII and *Bam*HI restriction enzymes as described in steps 1–3.

(6) The final recombinant plasmid was introduced to *B. anthracis* A16R for observation of green fluorescence.

Supplementary Figures

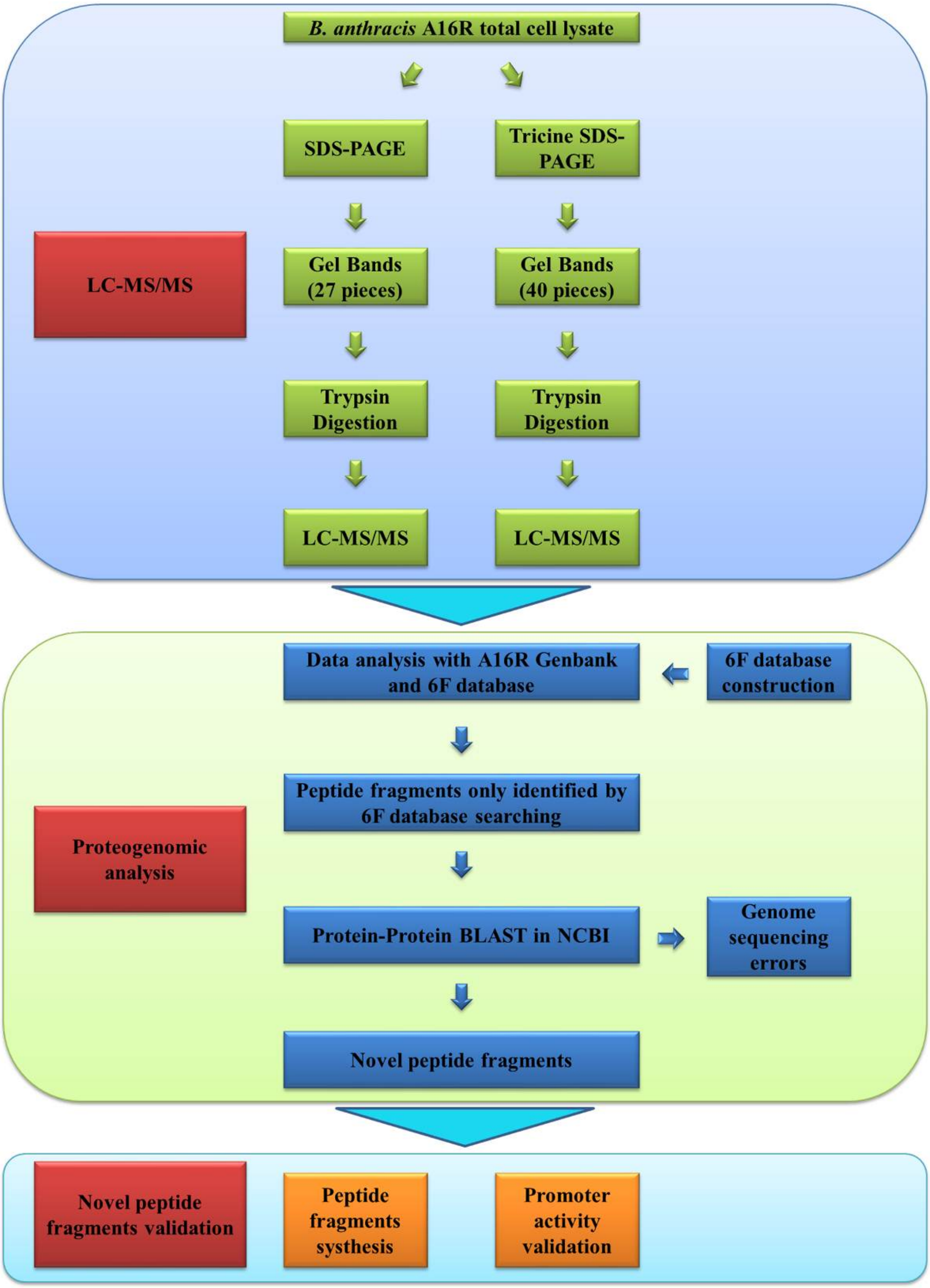

Figure S1. Experimental flow chart.

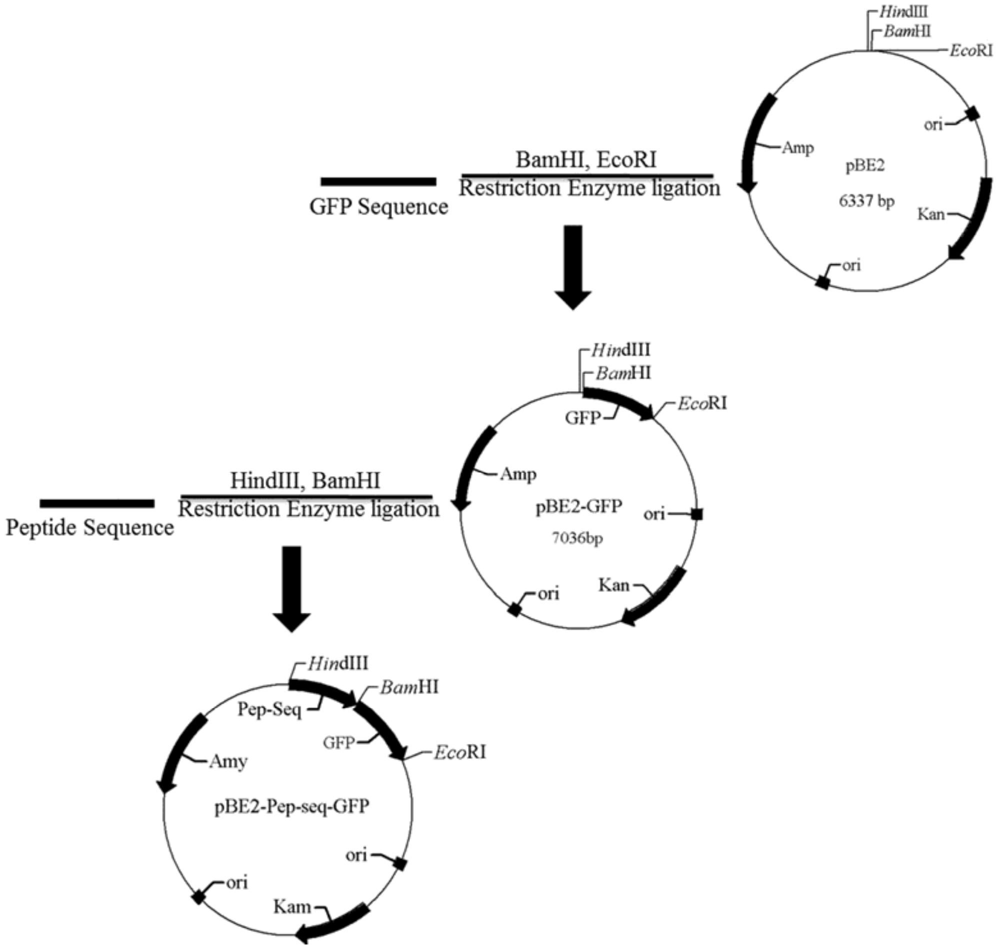

Figure S2. Vector construction.

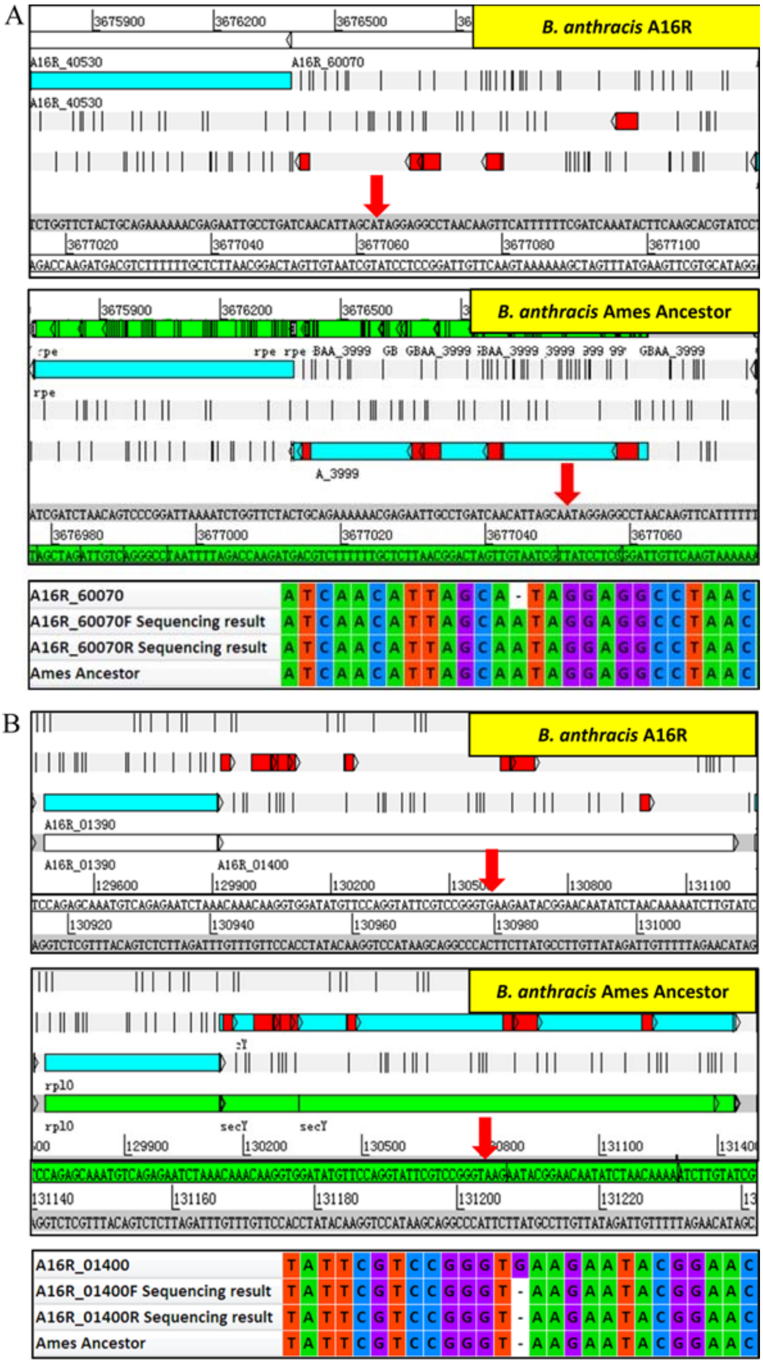
A
B. anthracis A16R
A16R_40530
A16R_60070
B. anthracis Ames Ancestor
A_3999
A16R_60070
A16R_60070F Sequencing result
A16R_60070R Sequencing result
Ames Ancestor
B
B. anthracis A16R
A16R_01390
A16R_01400
B. anthracis Ames Ancestor
rpl0
secY
A16R_01400
A16R_01400F Sequencing result
A16R_01400R Sequencing result
Ames Ancestor

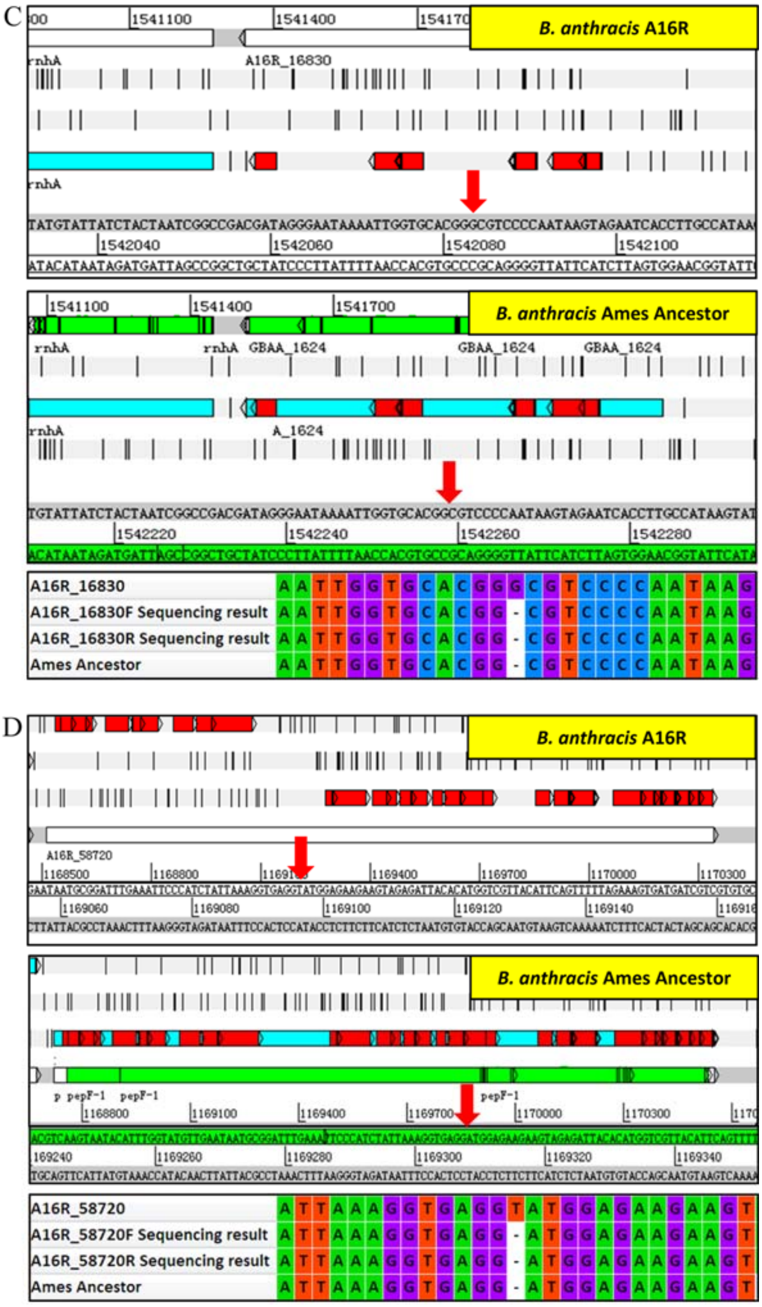

C
B. anthracis A16R
A16R_16830
rnhA
B. anthracis Ames Ancestor
GBAA_1624
A16R_16830
A16R_16830F Sequencing result
A16R_16830R Sequencing result
Ames Ancestor
D
B. anthracis A16R
A16R_58720
B. anthracis Ames Ancestor
pepF-1
A16R_58720
A16R_58720F Sequencing result
A16R_58720R Sequencing result
Ames Ancestor

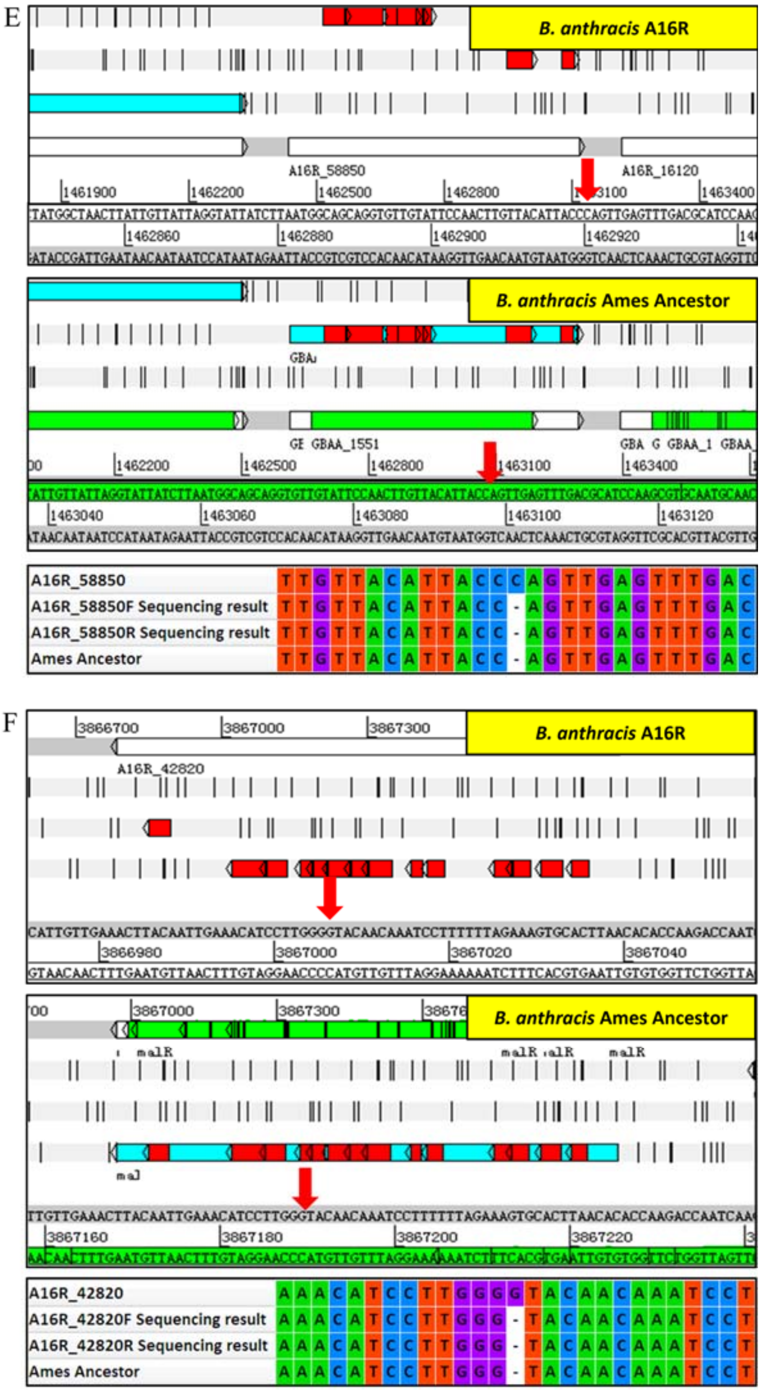

Figure S3. Genome sequencing error validation. Upper panel: Genomic annotation in *B. anthracis* A16R; Middle panel: Genomic annotation in *B. anthracis* Ames Ancestor; Lower panel: Sequencing validation. The red arrows represent the locations of peptides identified in this study.

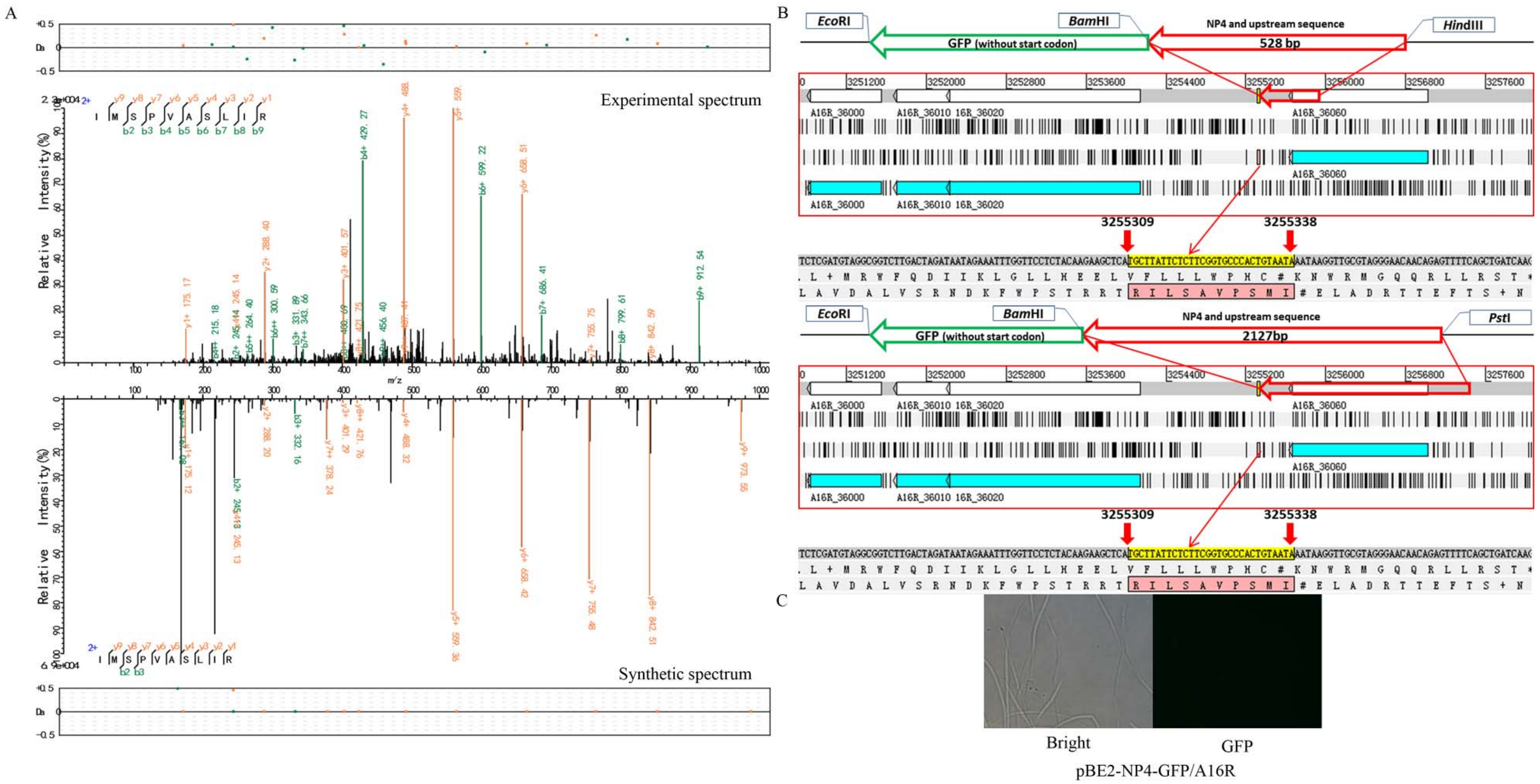

Figure S4. New peptide NP4. (A) Spectra of peptide NP4. (Top) Spectra filtered by proteogenomic analysis. (Bottom) Spectra of synthesis peptide. (B) The

encoded region of peptide NP4. The peptide NP4 is not annotated by current annotation. (C) GFP signal validation of NP4. No green fluorescence was observed when target fragments were inserted to the upstream of *gfp* gene.

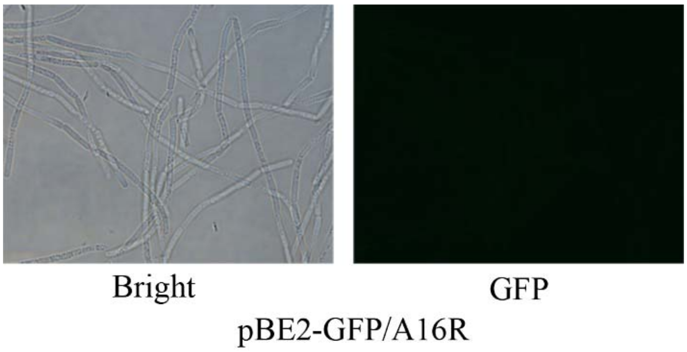

Figure S5. Observation of pBE2-GFP/A16R.

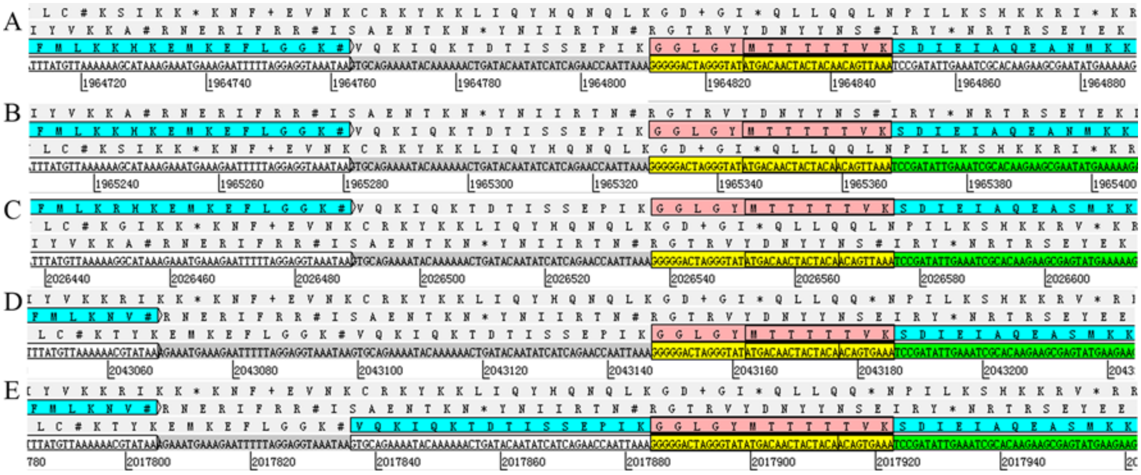

Figure S6. NP2 and corresponding annotations in *B. anthracis* and *B. cereus*.

(A) *B. anthracis* A16R, GI: 570710095.

(B) *B. anthracis* Ames Ancestor, GI: 50196905 (identical to strain A16R).

(C) *B. cereus* 03BB102, GI: 225862057 (identical to strain A16R and strain Ames Ancestor).

(D) *B. cereus* ATCC 14579, GI: 30018278. There is a frameshift due to the insertion of base “A” in the upstream gene compared to the above three strains. The annotation of the downstream gene is the same as in *B. anthracis*.

(E) *B. cereus* B4264, GI: 218230750. There is a frameshift identical to strain ATCC 14579, while the annotation of the downstream gene was different. The identification of peptide NP2 expression allowed for annotation correction.

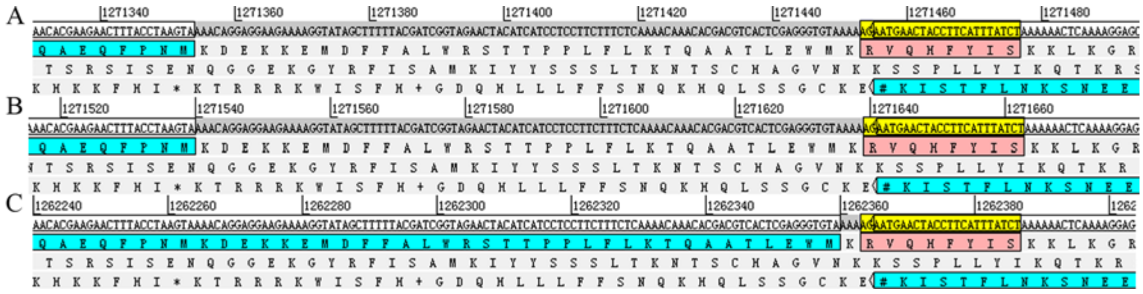

Figure S7. Annotations of NP3 are different in different *B. anthracis* strains. The initiation site annotated in *B. anthracis* H9401 is approximately 100 bp ahead of the other strains. (A) *B. anthracis* A16R. (B) *B. anthracis* Ames Ancestor. (C) *B. anthracis* H9401.

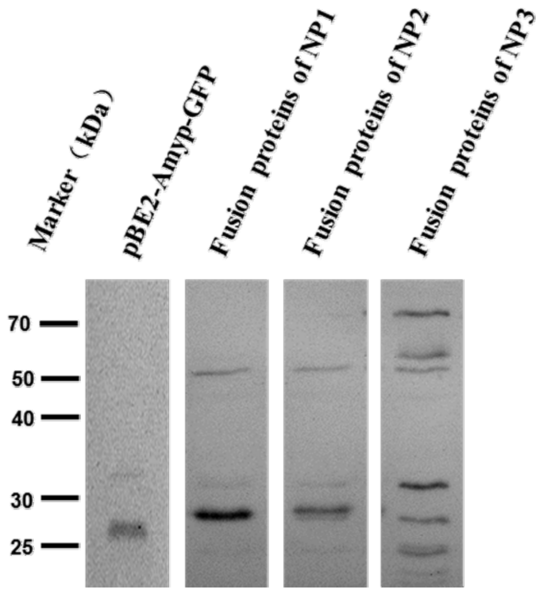

Figure S8. Western blot analysis to analyze the putative MWs of the fusion proteins constructed in this study.

Supplementary Table

Table S1 Selected key functional proteins identified in this study

Protein	gene	Description	MW (kDa)	p*I*	Unique peptides by Tricine SDS-PAGE	Total peptides by Tricine SDS-PAGE	Unique peptides by SDS-PAGE	Total peptides by SDS-PAGE
**Regulatory proteins**								
A16R_40180	*codY*	transcriptional repressor CodY	28.76	5.09	20	326	10	66
A16R_01030	*σH*	RNA polymerase factor sigma-70	25.23	5.14	14	49	11	21
A16R_44450	*spo0A*	chemotaxis protein CheY	29.39	7.20	20	186	7	36
A16R_56730	*plcR*	transcriptional regulator	25.40	7.70	7	14	2	2
A16R_61320	*atxA*	AtxA	55.53	9.11	16	148	9	53
A16R_61375	*pagR*	ArsR family transcriptional regulator	11.55	9.59	7	8	0	0
**Spore associated proteins**								
A16R_58640	-	SigE-dependent sporulation protein	7.87	4.61	1	1	0	0
A16R_15520	*spmA*	spore maturation protein	21.74	9.85	3	5	1	1
A16R_15530	-	spore maturation protein	18.79	6.54	1	1	0	0
A16R_49470	-	spore protein	13.98	5.81	1	1	0	0
A16R_49530	-	spore protein	6.81	6.13	1	1	0	0
A16R_03970	-	spore coat protein	19.38	5.31	2	2	0	0
A16R_13000	*rfbD*	spore coat protein	32.39	6.46	1	1	0	0
A16R_13040	*cotZ-1*	spore coat protein	16.14	4.95	2	3	0	0
A16R_13080	*cotZ-2*	spore coat protein	16.83	4.71	2	3	0	0
A16R_21090	*cotH*	spore coat protein	42.52	5.52	1	1	1	2
A16R_26000	-	spore coat protein	15.48	4.38	1	1	0	0
A16R_39580	*cotE*	spore coat protein	20.39	4.39	2	3	0	0
A16R_47490	-	spore coat protein CotS	41.11	5.85	1	2	0	0
A16R_52700	*cotS*	spore coat protein CotS	40.09	5.72	1	1	0	0
A16R_21080	-	Spore coat protein G	22.19	9.28	2	6	1	1
A16R_00640	-	spore cortex biosynthesis protein	26.05	10.54	1	1	0	0
A16R_44450	*spo0A*	chemotaxis protein CheY	29.39	7.20	20	186	7	36
A16R_58190	*soJ*	sporulation initiation inhibitor Soj	27.36	5.20	8	73	5	21
A16R_14210	*kinB-1*	sporulation kinase	45.32	9.07	1	1	0	0
A16R_15390	-	sporulation kinase	44.20	6.21	1	1	0	0
A16R_42780	*kinB-3*	sporulation kinase	47.75	7.63	8	29	1	2
A16R_02690	-	sporulation protein	36.10	9.19	1	1	0	0
A16R_16090	-	sporulation protein	30.45	9.01	2	2	0	0
A16R_28230	-	sporulation protein	50.00	7.67	2	2	0	0

A16R_29860	-	sporulation protein	5.79	9.60	1	2	0	0
A16R_47320	-	sporulation protein	21.61	5.35	4	6	0	0
A16R_29850	-	sporulation protein	5.79	9.87	1	2	0	0
A16R_23730	-	sporulation protein SpoOM	28.84	4.87	26	199	14	58
A16R_54580	-	sporulation regulator WhiA	36.31	9.28	15	19	3	4
A16R_41010	*sigE*	sporulation sigma factor SigE	27.62	7.74	3	6	0	0
A16R_43480	*sigF*	sporulation sigma factor SigF	29.16	5.31	7	8	2	2
A16R_41000	*sigG*	sporulation sigma factor SigG	29.75	6.13	2	3	0	0
A16R_00370	-	stage 0 sporulation protein	31.41	5.38	16	53	7	10
A16R_17140	-	stage 0 sporulation regulatory protein	8.49	9.44	2	5	0	0
A16R_56040	*spoIID*	stage II sporulation protein D	37.32	9.35	1	1	0	0
A16R_31710	-	stage II sporulation protein P	43.02	8.22	1	1	0	0
A16R_25480	-	stage II sporulation protein SB	28.36	7.98	2	2	0	0
A16R_25540	-	stage III sporulation protein AA	6.68	9.52	1	1	0	0
A16R_44670	*spoIIIAA*	stage III sporulation protein AA	34.37	7.61	1	1	0	0
A16R_44660	*spoIIIAB*	stage III sporulation protein AB	19.86	6.92	1	1	0	0
A16R_44630	*spoIIIAE*	stage III sporulation protein AE	42.17	8.69	1	1	1	1
A16R_44610	*spoIIIAG*	stage III sporulation protein AG	24.82	8.80	2	2	0	0
A16R_44600	-	stage III sporulation protein AH	24.62	8.96	1	3	0	0
A16R_15910	*spoIVA*	stage IV sporulation protein A	55.53	4.69	2	2	1	1
A16R_43420	*spoVAD*	stage V sporulation protein AD	36.36	5.15	1	2	0	0
A16R_43390	*spoVAF*	stage V sporulation protein AF	55.03	5.92	3	3	0	0
A16R_41130	-	stage V sporulation protein D	70.09	8.93	1	1	0	0
A16R_08600	*spoVR*	stage V sporulation protein R	56.16	5.46	2	2	0	0
A16R_22170	-	stage V sporulation protein S	9.41	10.27	6	24	0	0
A16R_39640	-	stage V sporulation protein S	8.86	6.18	27	219	0	0
A16R_32180	*gerSB*	spore germination protein	34.29	9.47	1	1	0	0
A16R_32190	*gerAA*	spore germination protein	48.81	8.73	3	5	0	0
A16R_36820	*gerSA*	spore germination protein	56.88	5.48	1	1	0	0
A16R_61360	-	spore germination protein	36.95	9.74	2	2	0	0
A16R_36830	*gerSB*	spore germination protein	42.01	9.51	1	1	1	1
A16R_32170	-	spore germination protein GerA	24.49	8.17	1	1	1	1
A16R_50460	*gerHC*	spore germination protein GerH	40.63	9.55	1	1	0	0
A16R_06990	*gerKC*	spore germination protein GerK	42.25	9.63	1	2	1	1
A16R_07020	*gerKA*	spore germination protein GerK	56.39	5.90	1	1	0	0
A16R_07770	*gerLA*	spore germination protein GerLA	54.51	6.48	2	2	0	0
A16R_12190	*gerPC*	spore germination protein GerPC	24.28	4.78	1	1	0	0
A16R_12160	-	spore germination protein GerPF	7.35	3.90	1	1	0	0
A16R_24020	-	spore germination protein GerPF	7.35	4.04	1	1	0	0
A16R_57200	-	spore germination protein GerQ	16.29	5.27	2	3	0	0
A16R_61355	-	spore germination protein XB	55.11	7.60	1	1	0	0
**S-layer proteins**								

A16R_09760	*sap*	S-layer protein Sap	81.57	6.54	80	3543	76	2184
A16R_09780	*eag*	S-layer protein EA1	91.31	5.70	90	4013	73	2576
A16R_33930	-	S-layer protein	40.31	5.84	31	530	22	171
A16R_10630	-	S-layer protein	64.33	8.65	14	24	5	5
A16R_11690	-	S-layer protein	38.24	9.08	3	39	3	38
A16R_12000	-	S-layer protein	24.81	7.03	11	41	9	35
A16R_12020	-	S-layer protein	24.80	7.84	11	39	7	11
A16R_12030	-	S-layer protein	23.61	4.81	8	37	6	17
A16R_19860	-	S-layer protein	37.49	6.60	2	35	2	2
A16R_23800	-	S-layer protein	42.79	6.98	3	26	1	1
A16R_28790	-	S-layer protein	50.34	8.77	0	0	1	1
A16R_37420	-	S-layer protein	55.43	5.64	2	7	1	1
A16R_51130	-	S-layer protein	29.85	9.26	11	38	4	5
A16R_61210	-	S-layer protein	76.16	9.16	9	19	7	8

Table S2 Entries in 6F database unmatched to Genbank annotation

	Protein in 6F database	MW	p*I*	Unique peptides by Tricine SDS-PAGE	Total peptides by Tricine SDS-PAGE	Unique peptides by SDS-PAGE	Total peptides by SDS-PAGE	Alignment in *B.anthracis* Ames Ancestor
1	A16R_1_00983	5.09	9.66	2	24	0	0	GBAA_0048
	A16R_2_01189	48.47	5.60	16	159	5	46	
2	A16R_1_02153	71.24	9.08	48	1386	15	233	GBAA_0103
	A16R_2_02493	70.71	9.19	43	1014	18	318	
3	A16R_1_02504	6.27	9.90	1	1	0	0	GBAA_0130
	A16R_2_02874	44.94	9.59	8	78	3	22	
	A16R_3_02780	12.32	9.84	1	10	1	7	
4	A16R_rv1_99783	18.71	7.80	5	9	1	1	GBAA_0225
	A16R_rv2_99279	21.93	7.13	2	8	1	2	
5	A16R_1_06101	39.16	8.60	24	238	11	74	GBAA_0290
	A16R_3_06204	15.55	5.40	8	39	4	31	
6	A16R_rv1_94422	123.41	5.78	38	469	7	45	GBAA_0530
	A16R_rv2_94146	6.11	8.79	3	16	0	0	
	A16R_rv3_93308	29.18	6.05	8	66	3	4	
	A16R_rv3_93408	11.71	5.12	3	41	2	11	
7	A16R_1_11294	6.14	9.70	2	9	1	6	GBAA_0544
	A16R_2_11321	19.43	6.23	7	35	5	16	
8	A16R_rv1_88995	13.21	5.14	2	15	0	0	GBAA_0785
	A16R_rv3_88605	49.58	5.42	9	80	3	9	
9	A16R_rv2_89071	24.82	9.28	6	17	5	13	GBAA_0796
	A16R_rv3_88408	22.28	9.18	5	10	2	3	
10	A16R_1_17879	18.58	6.09	6	10	4	10	GBAA_0875
	A16R_3_18244	39.93	5.16	11	40	4	6	
11	A16R_rv1_87316	31.37	5.47	15	71	13	60	GBAA_0881
	A16R_rv3_86977	3.84	4.95	2	3	0	0	
12	A16R_1_18255	6.42	9.35	4	41	3	48	GBAA_0885
	A16R_3_18479	82.85	6.60	65	3204	65	1919	
13	A16R_1_11942	13.03	9.95	1	1	0	0	GBAA_0924
	A16R_2_11982	21.31	9.28	1	1	0	0	
14	A16R_1_23977	24.84	4.82	10	238	2	43	GBAA_1206
	A16R_2_24901	7.25	7.96	3	57	1	11	
	A16R_3_24468	46.17	5.72	27	565	15	226	
15	A16R_rv1_81966	63.70	6.00	5	6	0	0	GBAA_1239
	A16R_rv2_82088	17.70	8.59	1	1	0	0	
16	A16R_2_27759	30.31	5.20	11	229	4	39	GBAA_1371
	A16R_3_27297	51.23	5.34	29	318	17	199	
17	A16R_1_29929	25.21	7.19	7	68	3	25	GBAA_1551
	A16R_2_30930	8.63	5.23	2	43	1	3	

18	A16R_2_31880	18.12	4.84	4	6	2	2	GBAA_1606
	A16R_3_31533	16.78	7.27	3	11	1	1	
19	A16R_rv1_75877	30.16	5.00	10	51	3	6	GBAA_1624
	A16R_rv3_75392	8.86	8.84	2	6	3	4	
20	A16R_1_39186	24.50	4.66	4	19	3	4	GBAA_1985
	A16R_3_39396	16.17	4.90	8	48	7	16	
21	A16R_rv1_69718	23.73	4.56	7	111	7	27	GBAA_1998
	A16R_rv3_68990	7.61	8.91	5	41	3	6	
22	A16R_rv1_35405	20.53	5.03	1	1	0	0	GBAA_2222
	A16R_rv2_34837	16.36	9.28	6	14	3	3	
23	A16R_1_45910	57.45	6.60	22	147	15	46	GBAA_2345
	A16R_2_45802	36.46	9.45	15	215	12	73	
24	A16R_rv1_27665	14.05	6.21	2	4	0	0	GBAA_2353
	A16R_rv3_26928	19.01	5.31	1	1	0	0	
25	A16R_2_47707	20.50	4.77	16	107	5	35	GBAA_2441
	A16R_2_47708	15.12	4.56	6	33	3	5	
26	A16R_2_48401	16.55	7.88	2	2	3	3	GBAA_2479
	A16R_3_48953	8.62	5.08	1	1	0	0	
27	A16R_1_54364	38.27	6.79	5	5	0	0	GBAA_2737
	A16R_3_54609	20.74	8.98	1	1	0	0	
28	A16R_rv1_56156	7.27	4.62	1	1	0	0	GBAA_2752
	A16R_rv3_55301	17.39	6.07	4	6	0	0	
29	A16R_rv1_54569	5.22	9.31	2	3	0	0	GBAA_2847
	A16R_rv3_53742	15.69	5.47	1	2	0	0	
30	A16R_rv1_54168	6.85	5.26	1	1	0	0	GBAA_2867
	A16R_rv3_53411	35.98	6.46	1	1	0	0	
31	A16R_2_56506	27.09	5.24	6	26	5	9	GBAA_2899
	A16R_3_57087	22.86	8.53	12	70	5	22	
32	A16R_rv1_52531	22.14	6.65	2	3	0	0	GBAA_2954
	A16R_rv2_52573	22.93	5.47	4	9	3	3	
33	A16R_rv1_49636	87.05	5.30	6	10	3	9	GBAA_3116
	A16R_rv2_49295	15.90	6.96	1	2	2	2	
34	A16R_1_63400	24.78	8.85	1	1	0	0	GBAA_3271
	A16R_3_63510	15.54	8.12	1	1	0	0	
35	A16R_rv1_45388	7.24	9.82	1	14	1	1	GBAA_3321
	A16R_rv2_45065	35.83	5.23	17	58	11	35	
	A16R_rv3_43942	7.02	9.52	1	5	1	4	
36	A16R_rv3_43943	3.32	4.49	2	13	1	4	GBAA_3367
	A16R_rv3_43944	56.50	4.75	12	93	9	35	
37	A16R_rv1_39511	12.39	9.06	1	4	0	0	GBAA_3613
	A16R_rv3_39092	9.79	8.23	1	1	0	0	
38	A16R_rv2_36699	56.34	5.39	21	136	22	112	GBAA_3737
	A16R_rv3_36916	11.81	7.99	7	43	5	34	
39	A16R_1_17500	47.76	9.24	18	80	5	17	GBAA_3740

	A16R_rv3_36812	19.16	9.45	3	13	0	0	
40	A16R_rv1_34033	22.03	5.45	10	116	5	38	BA_3915*
	A16R_rv1_34044	16.02	5.09	5	52	2	2	
41	A16R_rv2_33024	35.81	6.57	13	143	11	66	GBAA_3926
	A16R_rv3_33009	25.44	7.12	15	125	6	13	
42	A16R_rv2_32495	17.40	8.55	4	6	3	9	GBAA_3947
	A16R_rv3_32669	21.68	5.79	7	14	4	9	
43	A16R_rv1_32866	30.34	9.31	13	155	8	65	GBAA_3971
	A16R_rv2_31972	51.97	7.89	18	190	7	49	
44	A16R_rv2_31698	106.07	5.47	14	30	2	2	GBAA_3986
	A16R_rv3_31960	31.46	5.07	5	11	1	1	
45	A16R_rv1_32361	21.66	9.10	9	74	7	17	GBAA_3991
	A16R_rv2_31627	14.75	5.39	6	30	2	6	
46	A16R_rv1_32195	25.39	5.15	6	18	2	4	GBAA_3999
	A16R_rv2_31453	12.76	9.30	1	1	0	0	
47	A16R_rv1_32103	27.11	5.20	10	48	7	10	GBAA_4004
	A16R_rv1_32104	7.46	6.42	2	7	3	3	
48	A16R_rv1_31649	32.80	8.01	1	3	0	0	GBAA_4029
	A16R_rv3_30899	19.97	10.11	1	1	0	0	
49	A16R_rv1_28717	4.32	8.19	4	27	2	6	GBAA_4196
	A16R_rv3_28076	16.57	9.12	15	83	6	9	
50	A16R_1_79147	41.24	5.88	23	654	7	85	GBAA_4218
	A16R_3_78952	52.01	5.34	24	571	16	194	
51	A16R_rv1_28271	34.53	9.01	11	18	4	11	GBAA_4226
	A16R_rv2_27351	9.22	7.94	1	1	0	0	
52	A16R_rv2_24150	8.39	10.84	1	8	1	1	GBAA_4405
	A16R_rv3_24269	28.45	5.46	19	205	9	50	
53	A16R_rv1_23550	7.03	4.45	6	47	2	22	GBAA_4515
	A16R_rv2_22383	38.05	5.02	13	164	4	30	
54	A16R_rv1_23535	52.39	6.63	14	19	3	4	GBAA_4516
	A16R_rv2_22377	20.23	6.37	3	4	1	1	
55	A16R_rv1_22964	23.47	5.10	2	2	0	0	GBAA_4555
	A16R_rv2_21577	12.80	4.78	1	2	0	0	
56	A16R_rv1_22416	13.90	9.30	8	33	2	2	GBAA_4591
	A16R_rv3_21285	7.38	6.11	2	11	2	3	
57	A16R_rv2_20842	16.10	7.80	4	24	1	2	GBAA_4601
	A16R_rv3_21052	21.27	5.32	5	18	5	6	
58	A16R_rv1_21972	35.71	9.28	13	84	11	38	GBAA_4612
	A16R_rv1_21973	4.61	6.86	1	3	1	4	
59	A16R_2_84587	49.62	9.02	10	22	3	6	GBAA_4628
	A16R_3_84933	13.82	9.99	2	8	1	1	
60	A16R_rv1_19313	49.87	5.33	39	985	11	301	GBAA_4754
	A16R_rv2_18069	18.23	8.75	12	282	9	96	
61	A16R_1_87624	7.57	4.74	2	32	1	4	GBAA_4768

	A16R_2_86841	13.20	8.94	10	36	3	14	
62	A16R_rv2_17516	26.74	5.18	1	2	0	0	GBAA_4781
	A16R_rv3_17755	35.90	9.54	3	9	0	0	
63	A16R_rv2_14972	39.38	5.42	22	210	11	75	GBAA_4938
	A16R_rv3_14854	16.98	8.92	11	124	6	45	
64	A16R_rv1_14326	30.63	7.96	19	349	14	110	GBAA_5017
	A16R_rv3_13330	17.06	4.56	4	135	4	44	
65	A16R_1_91096	33.46	5.51	21	322	11	173	GBAA_5019
	A16R_3_90999	28.68	6.35	18	389	6	123	
66	A16R_rv1_10408	8.17	5.31	4	27	4	28	GBAA_5217
	A16R_rv2_10052	25.98	4.79	19	274	9	127	
67	A16R_rv2_07570	36.89	5.77	24	375	7	102	GBAA_5365
	A16R_rv3_07130	21.65	4.45	13	55	7	41	
68	A16R_rv1_05190	26.32	9.47	10	110	10	75	GBAA_5497
	A16R_rv2_05159	22.99	8.56	9	117	11	85	
69	A16R_rv1_04055	29.72	5.14	26	710	7	142	GBAA_5547
	A16R_rv2_03920	26.60	5.23	24	491	7	40	
70	A16R_rv2_06621	22.97	4.77	8	30	5	10	GBAA_5572
	A16R_rv3_06249	20.21	7.23	7	32	2	4	
71	A16R_1_101743	12.35	6.33	2	2	3	4	GBAA_5673
	A16R_3_101465	37.37	5.82	2	2	3	3	
72	A16R_2_101562	8.37	9.46	1	5	0	0	GBAA_5676
	A16R_3_101509	51.39	9.00	2	10	0	0	

* BA_3915 is the *recA* Gene annotated in *B. anthracis* Ames but not annotated in *B. anthracis* Ames Ancestor. The *recA* gene contains a group I self-splicing intron[1].

Table S3 Primers for sequencing errors validation

Primer	Sequence	location in *B. anthracis* A16R
A16R_1F	TTGGCTCGCTTCTTTCAACTCTG	3676540-3676562
A16R_1R	TATGTGCAGCATGAAGAAGGGAT	3677209-3677231
A16R_2F	ACAGGAGGAAATGGTGGTTATGC	130661-130683
A16R_2R	ACCCATTTCAGTTTCAGCCTTCA	131389-131411
A16R_3F	GTGTCACGACGTTGGATTGTTCC	3682539-3682561
A16R_3R	AAGCATGGTATCCCGGTCTTACA	3683465-3683487
A16R_4F	ATCTGTAAATGATTCGCCTTCT	1541740-1541761
A16R_4R	TTGCCCTACTCCAAATACTAAA	1542610-1542631
A16R_5F	TAAAGGGAAACTCGGTGAC	1168662-1168680
A16R_5R	ACCAGATGAATATGCTCC	1169607-1169624
A16R_6F	GGGTATGACAGGAGCGGAAGT	1462539-1462559
A16R_6R	AGGTCTACTCCGAACAGC	1463452-1463469
A16R_7F	GCTACTTCAATCGCTCAA	3866411-3866428
A16R_7R	AACAAATCGCATTCATCG	3867249-3867266
A16R_8F	CGCTCGTCCGCACCTAAT	1878812-1878829
A16R_8R	CGCCCTTCTACTAACTATGT	1879659-1879678

Table S4 Hypothetical proteins identified

Protein	Description	MW	p*I*	Unique Pepties by Tricine SDS-PAGE	Total peptides by Tricine SDS-PAGE	Total peptides by SDS-PAGE	Total peptides by SDS-PAGE
A16R_00030	hypothetical protein	8.06	6.18	5	13	2	2
A16R_00260	hypothetical protein	8.93	9.63	4	7	1	1
A16R_00610	hypothetical protein	55.78	4.52	18	72	13	36
A16R_00620	hypothetical protein	10.32	9.74	6	13	3	6
A16R_00890	hypothetical protein	21.03	6.51	8	14	3	8
A16R_00940	hypothetical protein	40.91	5.10	13	142	4	27
A16R_01020	hypothetical protein	19.83	9.00	7	10	1	1
A16R_01790	hypothetical protein	53.73	5.63	18	76	13	35
A16R_01890	hypothetical protein	21.74	5.09	7	15	4	7
A16R_01940	hypothetical protein	13.14	10.30	1	1	1	1
A16R_02260	hypothetical protein	34.70	7.69	8	12	5	5
A16R_02390	hypothetical protein	62.97	5.76	3	4	2	2
A16R_02730	hypothetical protein	82.18	5.55	38	336	19	103
A16R_02740	hypothetical protein	18.38	9.49	1	1	1	2
A16R_02920	hypothetical protein	25.24	5.47	8	20	7	9
A16R_03480	hypothetical protein	44.78	6.03	22	103	15	47
A16R_03950	hypothetical protein	14.31	9.12	6	50	2	4
A16R_04040	hypothetical protein	14.93	5.19	1	1	1	1
A16R_04070	hypothetical protein	30.95	5.56	16	27	5	8
A16R_04260	hypothetical protein	11.79	4.82	8	17	4	4
A16R_04510	hypothetical protein	63.68	6.14	27	238	16	64
A16R_04520	hypothetical protein	41.65	5.37	36	383	25	134
A16R_04540	hypothetical protein	8.61	4.73	5	12	4	6
A16R_04560	hypothetical protein	17.77	9.30	11	120	15	109
A16R_04700	hypothetical protein	42.76	5.59	1	1	1	2
A16R_04710	hypothetical protein	7.14	4.27	3	11	1	4
A16R_05250	hypothetical protein	134.02	5.87	1	1	1	1
A16R_05260	hypothetical protein	6.24	4.46	1	2	1	1
A16R_05280	hypothetical protein	17.80	4.49	1	1	1	1
A16R_05620	hypothetical protein	12.25	4.80	4	5	3	3
A16R_05730	hypothetical protein	21.05	5.49	10	21	3	4
A16R_06050	hypothetical protein	12.34	8.71	5	7	2	2
A16R_06060	hypothetical protein	26.34	4.45	12	102	9	38
A16R_06700	hypothetical protein	10.74	4.89	7	19	1	2
A16R_06900	hypothetical protein	32.88	4.94	14	43	7	13
A16R_07730	hypothetical protein	26.91	6.76	6	20	2	3
A16R_08570	hypothetical protein	9.41	9.70	1	1	1	1
A16R_08750	hypothetical protein	16.59	5.09	6	33	3	16

A16R_09030	hypothetical protein	30.18	5.80	1	1	1	1
A16R_09410	hypothetical protein	41.46	5.79	9	18	6	6
A16R_09540	hypothetical protein	13.58	4.68	9	58	10	70
A16R_09680	hypothetical protein	17.76	4.62	5	7	4	7
A16R_09710	hypothetical protein	14.86	8.53	9	26	7	20
A16R_09790	hypothetical protein	45.81	6.27	14	25	3	7
A16R_09960	hypothetical protein	24.49	8.62	8	25	2	10
A16R_10050	hypothetical protein	23.68	6.78	1	1	2	2
A16R_10250	hypothetical protein	14.68	7.71	2	3	1	1
A16R_10500	hypothetical protein	62.53	5.07	16	63	9	26
A16R_10830	hypothetical protein	11.03	4.76	8	93	2	3
A16R_10870	hypothetical protein	5.41	10.00	11	43	3	4
A16R_10890	hypothetical protein	6.75	6.56	5	14	4	6
A16R_11190	hypothetical protein	21.57	5.33	5	11	2	2
A16R_11390	hypothetical protein	31.03	8.71	3	6	2	2
A16R_11500	hypothetical protein	52.20	4.44	23	236	9	80
A16R_11580	hypothetical protein	8.34	9.04	1	1	1	2
A16R_11640	hypothetical protein	26.76	5.46	6	13	2	2
A16R_11840	hypothetical protein	37.28	4.82	7	19	5	6
A16R_12230	hypothetical protein	33.44	6.84	12	20	7	15
A16R_12260	hypothetical protein	13.46	10.12	5	15	1	1
A16R_12360	hypothetical protein	18.38	9.15	17	151	12	39
A16R_12370	hypothetical protein	32.88	4.87	15	54	7	17
A16R_12760	hypothetical protein	34.36	5.90	2	3	2	2
A16R_12780	hypothetical protein	22.17	5.72	9	24	6	9
A16R_13120	hypothetical protein	19.86	6.75	19	367	12	50
A16R_13130	hypothetical protein	28.14	5.81	21	127	7	28
A16R_13440	hypothetical protein	13.69	5.32	1	2	1	1
A16R_13480	hypothetical protein	9.36	5.06	6	48	4	9
A16R_13560	hypothetical protein	29.81	6.94	3	4	4	7
A16R_14580	hypothetical protein	30.59	7.23	2	2	1	1
A16R_15110	hypothetical protein	49.27	9.35	3	4	1	1
A16R_15140	hypothetical protein	20.49	4.73	2	4	1	1
A16R_15210	hypothetical protein	10.16	4.67	11	82	4	19
A16R_15340	hypothetical protein	23.92	4.84	12	61	6	13
A16R_15400	hypothetical protein	33.32	5.71	3	4	1	1
A16R_15410	hypothetical protein	7.23	5.22	3	8	1	1
A16R_15460	hypothetical protein	29.30	8.85	11	27	4	11
A16R_15480	hypothetical protein	15.35	5.16	8	18	4	7
A16R_15490	hypothetical protein	19.12	5.12	5	16	3	12
A16R_15770	hypothetical protein	36.27	5.90	12	26	6	7
A16R_15900	hypothetical protein	27.30	6.91	12	31	9	11
A16R_16010	hypothetical protein	48.33	4.30	17	42	12	22
A16R_16020	hypothetical protein	21.42	6.71	6	9	4	4

A16R_16030	hypothetical protein	17.84	5.30	14	82	7	14
A16R_16260	hypothetical protein	6.57	7.24	10	18	5	6
A16R_16380	hypothetical protein	7.27	5.70	6	17	3	3
A16R_16580	hypothetical protein	10.02	9.37	7	29	3	3
A16R_16600	hypothetical protein	17.48	4.58	4	6	1	2
A16R_16850	hypothetical protein	25.76	5.09	1	1	2	2
A16R_16960	hypothetical protein	11.23	4.92	1	2	1	1
A16R_17280	hypothetical protein	17.69	4.96	12	25	8	14
A16R_17490	hypothetical protein	13.54	5.23	1	2	1	1
A16R_18070	hypothetical protein	14.44	4.37	8	45	1	3
A16R_18120	hypothetical protein	31.98	5.66	4	16	2	4
A16R_18340	hypothetical protein	15.53	5.12	7	8	1	1
A16R_18440	hypothetical protein	46.92	5.30	27	77	13	15
A16R_19300	hypothetical protein	78.65	5.27	7	7	2	4
A16R_19550	hypothetical protein	8.67	9.70	3	4	2	2
A16R_19730	hypothetical protein	22.67	8.82	6	12	4	13
A16R_20730	hypothetical protein	12.82	9.45	1	1	1	1
A16R_20740	hypothetical protein	20.61	5.76	10	17	3	3
A16R_20770	hypothetical protein	11.86	10.07	1	2	1	2
A16R_20780	hypothetical protein	14.55	4.46	6	15	1	2
A16R_20810	hypothetical protein	17.77	5.19	7	23	9	14
A16R_20830	hypothetical protein	36.81	5.88	6	7	3	4
A16R_21150	hypothetical protein	33.41	6.76	5	30	2	6
A16R_21210	hypothetical protein	22.54	6.22	3	4	1	1
A16R_21280	hypothetical protein	20.42	6.24	3	4	1	1
A16R_21360	hypothetical protein	15.41	7.95	7	51	4	19
A16R_21590	hypothetical protein	7.17	10.66	1	1	1	1
A16R_21700	hypothetical protein	20.12	4.83	3	3	1	1
A16R_22330	hypothetical protein	15.96	4.89	10	37	4	13
A16R_22460	hypothetical protein	28.40	5.30	7	48	3	11
A16R_22470	hypothetical protein	29.60	5.06	6	27	6	9
A16R_22480	hypothetical protein	14.06	4.41	7	12	6	15
A16R_22560	hypothetical protein	16.60	4.26	5	11	2	2
A16R_22570	hypothetical protein	52.15	9.45	14	26	15	23
A16R_22580	hypothetical protein	13.54	4.47	6	17	7	8
A16R_22660	hypothetical protein	15.27	4.47	1	1	3	4
A16R_23480	hypothetical protein	70.96	5.95	2	2	2	2
A16R_23500	hypothetical protein	23.35	7.82	6	15	3	3
A16R_23540	hypothetical protein	14.80	6.29	5	14	3	6
A16R_23580	hypothetical protein	47.51	6.47	13	34	2	5
A16R_23650	hypothetical protein	10.58	4.40	2	5	4	9
A16R_23790	hypothetical protein	45.60	5.47	9	11	3	3
A16R_24030	hypothetical protein	12.70	6.41	7	17	2	3
A16R_24190	hypothetical protein	9.36	5.87	6	23	3	5

A16R_24250	hypothetical protein	6.88	7.32	1	2	1	2
A16R_24420	hypothetical protein	21.45	5.15	3	3	2	3
A16R_24580	hypothetical protein	24.49	4.36	4	6	5	5
A16R_24630	hypothetical protein	18.01	9.74	2	3	2	2
A16R_24750	hypothetical protein	36.97	8.94	2	15	1	1
A16R_24790	hypothetical protein	20.84	9.52	3	10	2	3
A16R_25020	hypothetical protein	23.21	6.06	11	44	4	6
A16R_25180	hypothetical protein	32.84	9.40	16	66	10	21
A16R_25570	hypothetical protein	17.63	7.90	5	33	5	13
A16R_26190	hypothetical protein	21.56	4.99	4	6	1	1
A16R_26300	hypothetical protein	14.82	5.20	5	6	1	1
A16R_26950	hypothetical protein	7.44	5.73	3	4	1	1
A16R_26970	hypothetical protein	38.60	4.90	5	7	4	5
A16R_27580	hypothetical protein	35.31	8.78	5	11	3	5
A16R_27620	hypothetical protein	42.24	5.90	6	12	7	9
A16R_27670	hypothetical protein	13.65	9.47	5	7	1	1
A16R_27780	hypothetical protein	15.09	7.81	7	8	2	3
A16R_28330	hypothetical protein	31.05	8.21	1	2	1	1
A16R_28680	hypothetical protein	16.64	6.24	6	10	2	3
A16R_28760	hypothetical protein	30.53	4.88	4	10	8	15
A16R_28980	hypothetical protein	17.10	7.65	5	10	2	2
A16R_29180	hypothetical protein	28.63	6.00	10	31	2	4
A16R_29290	hypothetical protein	15.92	5.33	4	12	3	10
A16R_29510	hypothetical protein	12.00	4.10	2	4	2	5
A16R_29550	hypothetical protein	24.14	5.71	4	6	3	3
A16R_29730	hypothetical protein	15.02	6.37	4	11	3	5
A16R_29890	hypothetical protein	31.74	5.27	9	25	6	16
A16R_29940	hypothetical protein	27.82	6.24	2	2	1	1
A16R_29990	hypothetical protein	17.98	5.15	5	11	4	10
A16R_30000	hypothetical protein	25.49	6.31	9	26	6	8
A16R_30020	hypothetical protein	17.28	8.46	4	4	1	2
A16R_30200	hypothetical protein	32.91	5.28	12	23	10	13
A16R_30380	hypothetical protein	19.91	7.07	8	14	3	6
A16R_30440	hypothetical protein	40.29	5.41	7	12	2	2
A16R_30630	hypothetical protein	18.92	5.12	3	3	2	2
A16R_30780	hypothetical protein	14.43	5.94	4	11	3	4
A16R_31130	hypothetical protein	24.56	6.05	12	118	8	43
A16R_31290	hypothetical protein	15.03	6.29	1	5	1	3
A16R_31330	hypothetical protein	16.33	8.44	12	19	5	5
A16R_31350	hypothetical protein	33.75	5.74	3	5	1	1
A16R_31380	hypothetical protein	35.86	9.00	1	1	1	1
A16R_31430	hypothetical protein	30.69	5.48	14	25	6	10
A16R_31730	hypothetical protein	18.49	4.94	4	9	1	2
A16R_32360	hypothetical protein	23.38	4.89	6	11	4	4

A16R_32550	hypothetical protein	13.65	5.42	5	7	3	5
A16R_32680	hypothetical protein	16.68	5.09	2	2	2	3
A16R_32710	hypothetical protein	50.75	4.96	19	115	4	4
A16R_32810	hypothetical protein	17.86	6.96	1	3	1	4
A16R_33310	hypothetical protein	26.00	6.70	1	1	1	1
A16R_33520	hypothetical protein	13.90	6.81	4	4	1	1
A16R_33750	hypothetical protein	47.75	4.93	21	120	12	47
A16R_33830	hypothetical protein	40.87	8.84	4	7	2	2
A16R_34070	hypothetical protein	21.12	5.38	9	14	4	4
A16R_34760	hypothetical protein	20.44	9.41	8	145	4	45
A16R_35210	hypothetical protein	31.29	5.25	4	11	3	3
A16R_35540	hypothetical protein	39.07	9.45	1	1	2	7
A16R_35810	hypothetical protein	13.30	6.19	7	22	2	2
A16R_35930	hypothetical protein	37.25	8.64	13	39	9	15
A16R_36200	hypothetical protein	55.13	5.24	22	39	5	8
A16R_36430	hypothetical protein	42.42	4.69	16	35	8	13
A16R_36790	hypothetical protein	13.00	6.39	5	14	2	2
A16R_37160	hypothetical protein	11.11	4.30	4	11	2	3
A16R_37170	hypothetical protein	16.26	6.16	7	13	2	7
A16R_37320	hypothetical protein	16.04	4.80	4	10	3	4
A16R_37410	hypothetical protein	7.91	9.27	2	3	1	1
A16R_37440	hypothetical protein	9.66	4.55	3	3	2	2
A16R_37470	hypothetical protein	28.80	9.62	1	1	2	2
A16R_37640	hypothetical protein	17.16	9.01	6	21	5	5
A16R_37920	hypothetical protein	8.37	10.36	11	187	6	23
A16R_38100	hypothetical protein	42.39	5.73	21	133	13	47
A16R_38120	hypothetical protein	12.52	9.62	2	12	1	1
A16R_38430	hypothetical protein	29.30	5.37	5	12	1	3
A16R_38440	hypothetical protein	37.64	6.14	13	39	7	11
A16R_38560	hypothetical protein	13.04	9.92	1	1	1	1
A16R_38830	hypothetical protein	46.01	5.18	14	67	10	22
A16R_39290	hypothetical protein	15.83	5.14	3	3	2	2
A16R_39720	hypothetical protein	30.40	5.28	9	44	3	5
A16R_39930	hypothetical protein	9.49	6.58	2	2	1	1
A16R_40370	hypothetical protein	13.19	4.66	8	15	2	4
A16R_40490	hypothetical protein	60.47	4.72	40	622	28	326
A16R_40500	hypothetical protein	13.13	5.59	10	67	3	6
A16R_40670	hypothetical protein	9.64	6.40	7	19	2	3
A16R_40680	hypothetical protein	34.14	5.62	12	18	4	7
A16R_40700	hypothetical protein	64.82	8.94	39	239	20	98
A16R_40970	hypothetical protein	25.26	5.94	10	43	4	5
A16R_41170	hypothetical protein	62.93	5.89	35	151	18	42
A16R_41190	hypothetical protein	17.93	5.09	8	58	9	44
A16R_41220	hypothetical protein	18.78	4.68	6	21	3	7

A16R_41230	hypothetical protein	22.28	6.62	3	5	6	8
A16R_41300	hypothetical protein	22.47	6.15	5	17	4	5
A16R_41350	hypothetical protein	78.02	6.33	1	1	1	1
A16R_41570	hypothetical protein	31.32	5.54	23	188	11	55
A16R_41860	hypothetical protein	42.05	8.50	8	10	4	4
A16R_41910	hypothetical protein	45.31	8.98	15	34	13	27
A16R_41920	hypothetical protein	37.39	5.68	11	32	7	24
A16R_42050	hypothetical protein	14.14	4.93	9	95	7	10
A16R_42160	hypothetical protein	10.74	4.82	6	23	3	5
A16R_42170	hypothetical protein	18.99	5.12	9	16	5	7
A16R_42180	hypothetical protein	49.90	5.51	22	130	14	48
A16R_42190	hypothetical protein	16.86	5.48	14	87	7	11
A16R_42210	hypothetical protein	10.89	5.19	6	14	2	4
A16R_42270	hypothetical protein	24.02	6.55	8	25	6	7
A16R_42280	hypothetical protein	10.85	5.13	4	6	2	2
A16R_42340	hypothetical protein	11.47	4.66	4	4	1	2
A16R_42350	hypothetical protein	15.03	8.57	6	22	2	5
A16R_42430	hypothetical protein	8.18	5.32	6	49	5	18
A16R_42540	hypothetical protein	9.11	4.36	6	87	1	2
A16R_42790	hypothetical protein	7.44	4.34	7	49	6	24
A16R_43010	hypothetical protein	28.69	5.42	2	2	2	2
A16R_43150	hypothetical protein	13.18	4.68	6	13	1	1
A16R_43330	hypothetical protein	20.44	8.68	6	11	5	8
A16R_43370	hypothetical protein	31.46	5.35	18	78	15	46
A16R_43630	hypothetical protein	27.52	6.44	19	98	12	46
A16R_44180	hypothetical protein	9.64	5.09	8	67	1	1
A16R_44200	hypothetical protein	39.59	5.10	33	450	26	321
A16R_44240	hypothetical protein	37.08	5.92	5	7	2	2
A16R_44290	hypothetical protein	16.04	5.21	9	43	3	8
A16R_44420	hypothetical protein	27.85	5.92	10	18	1	2
A16R_44570	hypothetical protein	14.07	4.70	11	273	8	55
A16R_44810	hypothetical protein	31.02	5.35	11	19	8	12
A16R_44830	hypothetical protein	12.58	9.92	9	73	2	9
A16R_45250	hypothetical protein	18.87	4.28	3	6	1	3
A16R_45360	hypothetical protein	22.92	4.72	4	6	1	1
A16R_45400	hypothetical protein	22.35	6.04	5	10	1	1
A16R_45520	hypothetical protein	28.70	5.32	13	105	7	63
A16R_45660	hypothetical protein	40.79	5.86	13	57	12	43
A16R_45870	hypothetical protein	16.46	7.88	12	40	10	23
A16R_46170	hypothetical protein	19.71	9.52	7	12	4	4
A16R_46620	hypothetical protein	8.41	6.04	4	16	7	15
A16R_46710	hypothetical protein	10.61	3.76	2	13	2	20
A16R_46730	hypothetical protein	10.32	5.78	7	28	4	6
A16R_46800	hypothetical protein	87.30	5.39	5	6	5	5

A16R_46810	hypothetical protein	24.72	4.40	6	15	8	11
A16R_46880	hypothetical protein	28.41	9.43	11	94	7	10
A16R_47340	hypothetical protein	12.35	4.88	3	7	2	2
A16R_47640	hypothetical protein	38.70	4.54	9	21	5	10
A16R_47660	hypothetical protein	23.58	5.88	23	336	15	79
A16R_48090	hypothetical protein	17.21	5.44	6	14	2	2
A16R_48140	hypothetical protein	17.41	5.54	10	28	7	22
A16R_48530	hypothetical protein	64.46	5.18	30	238	11	58
A16R_49140	hypothetical protein	48.73	6.54	19	36	9	19
A16R_49230	hypothetical protein	13.67	4.66	4	4	3	3
A16R_49250	hypothetical protein	12.68	4.74	3	4	2	2
A16R_49870	hypothetical protein	12.57	5.14	11	29	5	13
A16R_49910	hypothetical protein	15.50	9.58	4	18	1	1
A16R_49990	hypothetical protein	30.99	5.70	8	27	5	8
A16R_50040	hypothetical protein	32.76	5.88	14	78	12	48
A16R_50150	hypothetical protein	46.11	8.68	10	30	6	7
A16R_50190	hypothetical protein	16.13	5.57	6	18	3	5
A16R_50620	hypothetical protein	36.59	6.02	9	17	2	2
A16R_51000	hypothetical protein	8.55	8.64	1	1	1	2
A16R_51180	hypothetical protein	19.62	5.09	9	14	1	1
A16R_51340	hypothetical protein	26.87	4.35	3	6	1	1
A16R_51550	hypothetical protein	47.22	9.54	1	1	1	1
A16R_51750	hypothetical protein	27.81	5.09	17	243	12	100
A16R_52200	hypothetical protein	9.48	4.54	4	19	2	3
A16R_52480	hypothetical protein	16.59	4.86	2	3	3	3
A16R_52520	hypothetical protein	12.50	4.48	5	53	1	1
A16R_52710	hypothetical protein	18.22	5.14	7	29	7	18
A16R_52770	hypothetical protein	17.10	4.79	17	59	5	10
A16R_52810	hypothetical protein	12.01	5.67	8	14	1	1
A16R_53110	hypothetical protein	14.16	6.14	13	68	9	17
A16R_53330	hypothetical protein	11.19	7.99	4	8	1	2
A16R_53490	hypothetical protein	30.09	8.16	2	5	1	1
A16R_53720	hypothetical protein	12.73	4.78	5	6	2	2
A16R_53810	hypothetical protein	22.81	5.12	4	5	2	2
A16R_53890	hypothetical protein	16.19	4.59	12	277	12	92
A16R_54220	hypothetical protein	13.38	4.85	2	3	4	5
A16R_54330	hypothetical protein	48.43	5.43	2	3	1	1
A16R_54590	hypothetical protein	34.74	5.33	10	27	6	11
A16R_55070	hypothetical protein	23.94	5.50	14	53	4	5
A16R_55430	hypothetical protein	13.46	5.72	3	19	1	6
A16R_55440	hypothetical protein	73.42	6.03	5	6	1	2
A16R_55560	hypothetical protein	11.54	5.57	1	1	1	1
A16R_55620	hypothetical protein	18.90	9.27	3	3	1	1
A16R_55650	hypothetical protein	15.22	8.98	8	31	2	6

A16R_55880	hypothetical protein	104.06	6.30	53	227	11	21
A16R_55970	hypothetical protein	14.52	9.34	7	19	2	4
A16R_56360	hypothetical protein	20.73	5.57	8	28	4	6
A16R_56410	hypothetical protein	22.16	5.45	14	87	7	32
A16R_56590	hypothetical protein	19.36	4.71	8	23	7	14
A16R_56670	hypothetical protein	79.17	6.23	30	368	14	105
A16R_56910	hypothetical protein	16.42	7.02	6	17	1	1
A16R_57010	hypothetical protein	27.17	5.10	9	18	5	10
A16R_57030	hypothetical protein	19.01	7.03	11	49	7	21
A16R_57060	hypothetical protein	51.64	6.44	21	59	7	8
A16R_57120	hypothetical protein	8.13	8.66	3	4	1	1
A16R_57140	hypothetical protein	21.72	7.67	4	6	3	3
A16R_57980	hypothetical protein	32.64	5.95	3	4	1	1
A16R_58090	hypothetical protein	74.24	5.40	16	65	6	13
A16R_58150	hypothetical protein	7.91	9.12	6	19	1	2
A16R_58670	hypothetical protein	6.01	6.75	1	3	1	5
A16R_58815	hypothetical protein	7.75	9.51	1	1	1	1
A16R_59130	hypothetical protein	14.79	4.88	4	4	1	1
A16R_59305	hypothetical protein	7.51	9.92	1	8	1	4
A16R_59330	hypothetical protein	10.26	6.56	9	21	1	1
A16R_60550	hypothetical protein	8.42	9.57	2	4	1	1
A16R_60745	hypothetical protein	23.94	5.06	2	3	1	1
A16R_60785	hypothetical protein	26.87	8.64	9	77	4	14
A16R_60875	hypothetical protein	31.25	5.74	2	3	1	1
A16R_60955	hypothetical protein	6.90	9.87	1	1	1	1
A16R_60985	hypothetical protein	23.64	5.13	18	49	9	20
A16R_60995	hypothetical protein	44.47	9.42	1	1	1	5
A16R_61000	hypothetical protein	37.02	4.76	3	3	1	2
A16R_61105	hypothetical protein	49.80	8.66	10	12	5	8
A16R_61140	hypothetical protein	79.98	5.12	2	4	2	2
A16R_61325	hypothetical protein	18.22	8.35	10	22	3	4
A16R_61335	hypothetical protein	9.00	8.89	1	1	1	1
A16R_61435	hypothetical protein	19.92	6.87	4	4	6	6
A16R_61490	hypothetical protein	40.93	6.04	24	180	16	61
A16R_61500	hypothetical protein	56.61	8.71	21	58	13	21
A16R_61515	hypothetical protein	17.17	4.65	3	4	1	2

1 Ko, M., Choi, H. & Park, C. Group I self-splicing intron in the recA gene of Bacillus anthracis. *J Bacteriol* **184**, 3917-3922 (2002).
